# An evolutionary account of impairment of self in cognitive disorders

**DOI:** 10.1007/s10339-022-01110-4

**Published:** 2022-09-30

**Authors:** Antonio Benítez-Burraco, Ines Adornetti, Francesco Ferretti, Ljiljana Progovac

**Affiliations:** 1 Department of Spanish, Linguistics, and Theory of Literature (Linguistics), Faculty of Philology, University of Seville, Seville, Spain; 2 Cosmic Lab, Department of Philosophy, Communication and Performing Arts, Roma Tre University, Rome, Italy; 3 Linguistics Program, Department of English, Wayne State University, Detroit, USA

**Keywords:** Cognitive disorders, Notion of self, Human self-domestication, Aggression, Cross-modality

## Abstract

Recent research has proposed that certain aspects of psychosis, as experienced in, e.g., schizophrenia (SCZ), but also aspects of other cognitive conditions, such as autism spectrum disorders (ASD) and synesthesia, can be related to a shattered sense of the notion of self. In this paper, our goal is to show that altered processing of self can be attributed to an abnormal functioning of cortico-striatal brain networks supporting, among other, one key human distinctive cognitive ability, namely cross-modality, which plays multiple roles in human cognition and language. Specifically, our hypothesis is that this cognitive mechanism sheds light both on some basic aspects of the minimal self and on some aspects related to higher forms of self, such as the narrative self. We further link the atypical functioning in these conditions to some recent evolutionary changes in our species, specifically, an atypical presentation of human self-domestication (HSD) features. In doing so, we also lean on previous work concerning the link between cognitive disorders and language evolution under the effects of HSD. We further show that this approach can unify both linguistic and non-linguistic symptoms of these conditions through deficits in the notion of self. Our considerations provide further support for the hypothesis that SCZ and ASD are diametrically opposed cognitive conditions, as well for the hypothesis that their etiology is associated with recent human evolution, leading to a deeper understanding of the causes and symptoms of these disorders, and providing new cues, which can be used for an earlier and more accurate diagnostics.

## Introduction

Schizophrenia (SCZ) and autism spectrum disorders (ASD) are prevalent neurodevelopmental conditions often resulting in several highly distinctive cognitive and social characteristics, including deficits in language structure and use (Bailey et al. 1996; Tager-Flusberg et al. 2005; van Os and Kapur 2009; Stephane et al. 2014). Both conditions emerge from a complex interaction among genetic alterations, epigenetic changes, and environmental factors, resulting in an atypical development and wiring of selected brain pathways during growth, which in turn impacts upon diverse cognitive functions and ultimately results in their distinctive neurocognitive and behavioral profiles (Li et al. 2009; Stefanatos and Baron 2011; Bakhshi and Chance 2015; Cannon 2015). Crespi and Badcock (2008) pointed out that SCZ and ASD exhibit quite opposite features, from neurodevelopment to brain structure and function to cognitive abilities to behavior. Specifically, they noted that the two disorders can be regarded as mirror conditions for traits related to our social brain, including social cognition, gaze, and agency (hyperdeveloped in SCZ and underdeveloped in ASD). Ultimately, they hypothesized that these differences may result from opposite patterns of abnormal genomic imprinting. Ongoing research on the etiology of SCZ and ASD suggests that an altered notion of self could be a key factor accounting for many distinctive features of these two conditions and that with respect to this notion of self, SCZ and ASD can be regarded as mirror conditions, too. Accordingly, ASD seems to correlate with an inflated sense of self (e.g., Frith and Happé 1999; Lombardo and Baron-Cohen 2010; Lombardo et al. 2010), whereas a deflated, diminished sense of self can be posited for SCZ (e.g., Henriksen and Nordgaard 2014; Lysaker and Lysaker 2010). Altered notion of self is also found in other conditions of interest for our proposal, particularly, some forms of synesthesia (e.g., Banissy and Ward 2013; Cioffi et al. 2016). All three conditions present with altered patterns of cross-modality, which will be a central concern for us. We consider cross-modality to be one key feature of the human cognitive phenotype, the feature that enables one to transcend the boundaries of core cognitive systems (in the sense of Spelke 2000, 2003; Kinzler and Spelke 2007), thus allowing us, among other things, (i) to merge concepts and create new ones, (ii) to chunk syntactic pieces and form more complex syntactic structures, and (iii) to put into relation different domains, as attested in figurative uses of language.

In this paper, we will present evidence supporting the view that altered sense of self in conditions like SCZ, ASD, and synesthesia can be linked to an atypical presentation of specific innovations in the evolution of human cognition, particularly, cross-modality, and, that it might result, partly, from an altered manifestation of features of human self-domestication (HSD). According to the HSD hypothesis, several key aspects of the human distinctive phenotype are the outcome of an evolutionary process similar to animal domestication (Hare 2017). Under this view, different factors (e.g., the advent of co-parenting, the rise of community living, changes in our foraging ecology, and/or the deterioration of the human environment during the Last Glaciation) promoted selection in humans against reactive aggression and toward prosocial behavior, this resulting in physical, behavioral, and even cognitive changes that enhanced our social cognition, cooperation and social networks, and that paved the way toward our advanced technology and sophisticated culture (see Hare and Woods 2020 for details). Because domestication results in more complex communicative signals in some animal species, particularly, songbirds, HSD has been claimed to have favored the emergence of many structural features of modern languages through a cultural mechanism (Thomas and Kirby 2018; Benítez-Burraco and Progovac 2020), but also of modern uses of language, that is, modern pragmatics (Benítez-Burraco et al. 2021) (for a gene-culture feedback loop in this respect, see Benítez-Burraco and Progovac 2020, 2021). Interestingly, in line with the view that SCZ and ASD are diametrically opposed conditions of the brain, these two conditions lie as well on the opposite poles of the HSD spectrum, with SCZ showing some exaggerated features of HSD (Benítez-Burraco et al. 2017), and ASD showing certain attenuated features of HSD (Benítez-Burraco et al. 2016).

As we will show, the mechanism that links an altered presentation of HSD features in these conditions to an altered sense of self boils down to the management of aggression and its impact on cross-modality, which is at the core of the HSD hypothesis, and ultimately, to the inhibition/disinhibition in selected brain circuits. In Benítez-Burraco and Progovac (2021), we proposed that during human evolution, an enhanced connectivity between cortico-striatal networks emerged, firstly aimed at curtailing reactive physical aggression, but later aimed at accommodating simple grammars with metaphorical language, which relies heavily on the mechanism of cross-modality. Both processes (suppression of reactive aggression and cross-modality) clearly implicate cortico-striatal networks, and tend to cluster together in cognitive disorders. More specifically, our view is that both enhanced cross-modality and (the suppression of) reactive aggression, including verbal aggression, rely on a precise degree of (dis)inhibition of connectivity in the cortico-striatal brain circuits, the same circuits that are also essential for the processing of grammar, and language more generally. This entails that improved cross-modality, as observed in present-day humans, was (i) partly a result, or a side effect, of the HSD processes that initially favored specifically the dense connectivity in this network, enabling better control of reactive aggression by the higher, cortical structures of the brain, and (ii) partly a result of the cultural emergence of simple forms of language/grammar, relying heavily on metaphorical extension, which in turn contributed further to the connectivity of these brain networks, and to the reduction in reactive aggression.

We find these considerations relevant for the etiology of SCZ and ASD, as both conditions show altered management of aggression and altered processing of metaphorical (figurative) language. Hence, higher levels of reactive aggression are exhibited in ASD, in comparison with both typical and SCZ populations (Hill et al. 2014; Fitzpatrick et al. 2016). With regard to metaphorical language, ASD often exhibits difficulties in establishing connections between two elements of a compound which rely on stretching the meaning (e.g., Riches et al. 2012; Kambanaros et al. 2019), as well as in understanding abstract concepts and metaphors more generally (Dodd 2005: 47; Jordan 2010). People with SCZ can also be significantly impaired in the comprehension of novel metaphors (Rapp et al. 2018), as well as the comprehension of implicit information and humor (Pawełczyk et al. 2018). Nonetheless, SCZ individuals exhibiting a mild, nonclinical manifestation of psychotic-affective conditions in fact show relative strengths, e.g., when interpreting metaphors, emotions, humor, and irony (Crespi 2008: 238). In addition, relatives of people with SCZ seem to have an advantage in artistic expression and originality (Fink et al. 2014). It is also of note that SCZ presents with lower levels of reactive aggression relative to ASD, but higher levels of proactive (i.e., premeditated) aggression (Bo et al. 2013). In other words, while both conditions, ASD and SCZ, show deviation from the typical population, when compared with SCZ, ASD shows markedly higher reactive aggression. Overall, this constitutes some evidence for ASD and SCZ presenting with diametrically opposed symptoms in the domains of aggression management and figurative language, but also for milder or in-between conditions showing some adaptive advantages.^1^

Building on previous research (specifically, Benítez-Burraco and Progovac 2021), we contend that these opposite symptoms (in fact, opposite poles of a continuum) can be ascribed to atypical inhibition (ASD) vs. disinhibition (SCZ) of global connectivity of the brain, more specifically connectivity in selected cortico-striatal brain networks, which, as noted, are involved in the control of reactive aggression, but also in cross-modality. Accordingly, it has been reported that, more generally, individuals with ASD differ from neurotypical subjects by being biased toward local, at the expense of global, conceptual processing, leading to difficulties in generalizing information, i.e., to a weakened drive to detect meaning by looking at the “big picture” (Happé and Frith 2006). This cognitive processing pattern observable in people with ASD, which has been termed *weak central coherence* by Frith (1989), is linked to the concepts of *field-independence/field-dependence* introduced by Witkin et al. (1962) to classify individual differences in visual scene processing. An individual who is field-independent is more prone to perceive an element independently from its global context; in contrast, field-dependent people are highly influenced by the globality of the visual scene when processing its features.^2^ Weak central coherence can be ascribed to reduced cross-modality, a result of inhibited global connectivity (Watanabe and Rees 2016; Van Leeuwen et al. 2019). At the same time, individuals on the ASD spectrum often show increased local connectivity. As discussed below, this characterization of ASD can shed light, specifically, on the nature of the altered sense of self found in people on the spectrum, but also on the lack of global coherence in their narratives. By contrast, SCZ seems to involve a profound failure of inhibition of global connectivity. In particular, Silbersweig and colleagues (1995) ascribe SCZ hallucinations in both auditory and visual modalities to the abnormal disinhibition of cortical–subcortical circuits, which, as noted, are also involved in the management of aggression (see also Sterzer et al. 2018; Van Leeuwen et al. 2020, for alternative views of hallucinations in SCZ). Van den Berg and colleagues (2012) offer a more nuanced, dynamic model for the mechanism for schizophrenia (see also Rentzeperis and van Leeuwen 2021), where they conclude that, after initial fragmentation, there is ultimately a shift in the balance from local to global functional connectivity.

Interestingly, synesthesia can be characterized as exhibiting a combination of both increased local connectivity between sensory brain regions and enhanced global connectivity (Hubbard 2007; Hubbard and Ramachandran 2005). Synesthesia is not typically considered a disorder, but a condition in which stimulation of one sensory or cognitive pathway leads to automatic experiences in another sensory or cognitive pathway (Cytowic 2002; Cytowic and Eagleman 2009). According to Grossenbacher and Lovelace (2001), synesthesia is a result of disinhibited cortical sensory feedback (see also Cytowic 1993, who emphasizes limbic mediation and cortical depression as relevant for synesthesia). One proposed explanation for this hyper-connectivity in synesthesia invokes a failure of adequate pruning of excessive brain connections both locally and globally, the pruning that typically takes place in ontogeny (e.g., Maurer 1993; Baron-Cohen et al. 1993; Ramachandran and Hubbard 2001; Maurer and Mondloch 2006; Ward 2013). Significantly, ASD has been characterized as a condition resulting from over-pruning of selected brain circuits, i.e., from aggressive synaptic pruning during childhood (Thomas et al. 2016). Interestingly, a key etiological factor of SCZ seems to be an aberrant synaptic pruning in selected brain areas, particularly, the prefrontal cortex during adolescence (Woo 2014).

These differences in local vs. global connectivity, and ultimately in excitatory vs. inhibitory synapses, apparently explain both the shared features and the differences between ASD, SCZ, and synesthesia, for example, why both ASD and synesthesia are advantageous for the attention to detail (correlated with enhanced local connectivity), whereas only synesthesia, as well as mild cases of SCZ, seems to also show advantages in creativity, metaphoricity, and imagination (correlated with enhanced global connectivity). In other words, both the vulnerabilities and the advantages exhibited by individuals with these conditions seem to result from an altered pattern of inhibition/disinhibition of brain connectivity, either locally, or globally, or both, with trade-offs in systematicity and attention to detail, vs. creativity and imagination, the two sides acting as poles of otherwise continuous variability attested in humans. On this view, there is a dichotomy between perceiving the world as hyper-real (ASD-like) versus surreal (SCZ-like), where synesthesia seems to fall somewhere midway. A similar pattern emerges, as noted, with regard to social functions, including aggression. In the next section, we rely on the findings and considerations outlined above to revisit the notion of self in ASD, SCZ, and synesthesia. We provide new evidence in favor of the hypothesis that ASD and SCZ represent two opposite poles of the social brain, specifically when it comes to the notion of self, this ultimately correlated with their opposite positions within the HSD spectrum. This construal can account for some of the peculiarities of the sense of self typically observed in these two conditions.

## The notion of self in SCZ, ASD and synesthesia

SCZ and ASD have long been described as disorders of the self (Bleuler 1911/1950; Kanner 1943; Asperger 1944). Bleuler (1911/1950) suggests that SCZ is linked to an alteration in a person’s basic sense of self—“the personality loses its unity” (p. 9)—stressing that persons with this disorder struggle to recognize themselves as subjects of their own experiences and agents of their own actions. In his famous early description of the children with autistic disturbances of affective contact, Kanner (1943) mentioned their difficulties in maintaining a constant self-concept and problems in adapting it to changing environment. A large body of research has linked some aspects of the impaired sense of self in SCZ and ASD to the atypical functioning of social cognition observable in these conditions, including aberrant functioning of the Theory of Mind (ToM) system (e.g., Frith & Happé 1999; Lysaker et al. 2011; Lyons and Fitzgerald 2013; Tordjman et al. 2019; Arnaud 2020). These aspects contribute to shape the dimension of the self that has been termed *interpersonal self* (Zahavi 2010): “It is the result of one’s social interactions with other people, as well as one’s ability to relate to other people. This dimension encompasses basic ways of differentiating oneself from others … and more complex characteristics attributed to oneself, such as the way one appears to others, … empathic or egocentric personality, openness to others and propensity to help” (Arnoud 2020, 3–4). Nonetheless, the concept of self is difficult to define. Different models have been proposed to account for its nature (e.g., Dennett 1991; Gallagher & Shear 1999; Gallagher 2000; Dehaene 2014; Zahavi 2014; Di Francesco et al. 2016). The difficulty of providing a definition of this concept arises from the fact that the self is a multifaceted entity entailing different functions, including (i) self-recognition (Meltzoff 1990; Lewis & Ramsay 2004), (ii) body awareness (Mehling et al. 2009), (iii) sense of ownership (Gallagher 2000; Tsakiris et al. 2007), (iv) sense of agency (Gallagher 2000; Haggard 2017), (v) self and other differentiation (Jeannerod 2007), (vi) awareness of other minds (Frith 2002; Decety & Chaminade 2003), and (vii) awareness of being the same person across the time (Gallagher 2000; Habermas & Köber 2014). Given the multiple functions constituting the self, we expect that its cognitive underpinnings are multiple, too, going beyond ToM and including (but not limited to) social cognition (e.g., Uddin et al. 2007), executive functions (e.g., Hofmann et al 2012), and mental time travel (MTT), the cognitive system allowing to mentally project backward and forward in time (e.g., Quoidbach et al. 2008; Cosentino 2011; Adornetti & Ferretti 2021).

Below, we provide a brief characterization of how three central dimensions of the self ((i) ownership and (ii) agency, Sect. “Sense of ownership and sense of agency”., and (iii) self-other differentiation, Sect. “Self-other differentiation”.), as well as the underlying cognitive functions, manifest as altered in SCZ, ASD, and, to some extent, synesthesia. In this paper, we focus on aspects that can be attributed to atypical patterns of brain connectivity and inhibition/disinhibition.

### Sense of ownership and sense of agency

The sense of ownership and the sense of agency constitute what Gallagher and Zahavi (2000); Zahavi, (2010), (2014); Gallagher and Zahavi (2012) labeled *minimal self*, i.e., a self that is accessible to immediate self-awareness: “a consciousness of oneself as an immediate subject of experience, unextended in time” (Gallagher 2000, p. 15). The sense of ownership refers to the feeling that one’s body is one’s own (i.e., the sense that it is my body that is moving), whereas the sense of agency refers to the experience of controlling one’s own actions and, through them, the course of events in the outside environment (Haggard 2017). Along with other social–cognitive processes (i.e., joint attention, gaze monitoring, and the detection of intentional movements), it is considered to be an important developmental precursor of ToM (Abu-Akel 2003; Decety and Chaminade 2003). Russell (1996) proposed that an impairment in agency monitoring processes could impact upon the acquisition of ToM.

In this respect, SCZ exhibits abnormalities both in the sense of ownership (e.g., Peled et al. 2000; Ferri et al. 2014; Gallese and Ferri 2014) and in the sense of agency (e.g., Frith et al. 2000; Garbarini et al. 2016). Atypical body ownership in SCZ has been investigated through paradigms such as the Rubber Hand Illusion (Peled et al. 2000; Thakkar et al. 2011), which is an example of multimodal/multisensory integration, i.e., visuo-tactile integration, that depends on proprioceptive processing. Botvinick and Cohen (1998) found that neurotypical individuals who watch a rubber hand being stroked while one’s own unseen hand is being stroked simultaneously, often feel a sense of ownership over the rubber hand, along with a shift in perceived position of the real hand. Thakkar and colleagues (2011) explored this phenomenon in individuals with SCZ and found that they reported a stronger Rubber Hand Illusion than controls. This result supports the view that individuals with SCZ exhibit alterations in the multisensory integration relating to bodily self-experience. Interestingly, vividness of the illusion was associated with elevated hallucinations (in line with other studies, e.g., Peled et al. 2003), but also with delusions of reference and delusions of control.

Often some of these symptoms, such as acoustic-verbal hallucinations and delusions of alien control along with thought insertion, are interpreted as involving problems with the sense of agency, which is also altered in SCZ (Frith et al. 2000; Hur et al. 2014; Garbarini et al. 2016), leading to mistakes about the agency of various bodily movements (Frith & Done 1989; Gallagher 2000; Jeannerod 2009; Renes et al. 2013). Individuals who show these symptoms wrongly ascribe their experiences to other people with respect to agency, e.g., they state that someone else caused their actions. These represent attribution errors (*under-attribution*) and are the most frequently detected patterns in SCZ. The opposite pattern (*over-attribution*) may also be noted, i.e. the belief that they can control the thoughts and the behavior of other people (aka *megalomania*) (Jeannerod 2009, p. 530).

When it comes to ASD, contrasting results emerge. As for the sense of ownership, Paton et al. (2012) found that individuals with ASD experience the Rubber Hand Illusion to much the same degree as the control group. Cascio and colleagues (2012) investigated the phenomenon in children with ASD revealing that they were initially less susceptible to the illusion than the comparison group but showed the effects of the illusion after 6 min. Most studies investigating the sense of agency in ASD have reported no impairments in this process (e.g., Russell and Hill 2001; David et al. 2008; Williams & Happé 2009). On the other hand, Russell and Jarrold (1999) report that children with ASD had difficulties in correctly deciding whether an action had been produced by themselves or by another agent. All in all, we can conclude that the sense of agency is more affected in SCZ than it is in ASD.

As for synesthesia, research indicates that both the sense of ownership and the sense of agency are altered. For example, there is evidence that mirror-sensory synesthesia and responses to the Rubber Hand Illusion are related. In mirror-touch synesthesia (MTS), one of the most prevalent forms of synesthesia in which individuals feel tactile sensation on their own body by observing touch to other individuals (Blakemore et al. 2005; Banissy et al. 2009), the Rubber Hand Illusion occurs in the absence of synchronous tactile signals (Aimola-Davies and White 2013). An investigation by Cioffi and colleagues (2016) found that the sense of agency is also affected in MTS. In their study, the authors employed a vicarious agency paradigm: participants are placed in front of a mirror and an experimenter sits behind a curtain hidden from the participant’s view. The participants listen to action instructions and then observe an action performed by the experimenter with his/her hands. The hand gesture performed by the experimenter could be congruent or incongruent with the instructions. For example, in the mismatch condition, after the instruction “make a waving gesture” the experimenter snaps their fingers. Cioffi and colleagues (2016) showed that individuals with MTS had a higher sense of agency over the movements in the congruent condition compared to non-synesthete controls, suggesting that “difficulties in self-other processing may be a fundamental disturbance in MTS” (Cioffi et al. 2016, p. 429). The results of this study are also relevant to shed light on the relationship between the sense of agency and the sense of ownership. In fact, Cioffi and colleagues (2016) also evaluated to what degree the participants felt that the arm belonged to him/her. In line with previous research attesting an amplified sense of ownership in MTS (Aimola et al. 2013; Maister et al. 2013), they found that in individuals with MTS there was a stronger feeling of ownership toward the experimenter’s hands compared to controls in both match and mismatch conditions. According to Cioffi and colleagues (2016), this data suggests that in MTS “alterations in the sense of agency can be linked to more profound disturbances in the sense of ownership” (p., 426).

### Self-other differentiation

Classical developmentalists, such as Baldwin (1902), Cooley (1902), Mead (1934), as well as modern developmentalists, such as Stern (1985) and Neisser (1988), have stressed the role of other persons in the development of the awareness of self. As Hobson (1990) maintained: “an individual requires a concept of other persons as subjects of experience if he is to acquire a developed concept of self (…) Awareness of persons—that is, awareness of other individuated centers of subjective orientations toward the world—is a precondition for self-reflection and self-consciousness, for indexical thought, and ultimately for an objective view of the mind-independent world” (Hobson 1990, p. 155). The construction of reflexive forms of consciousness underlying the development of self seems to presuppose the recognition of others as persons. Within this theoretical framework, recognition of others as persons works as a mirror through which to look at oneself and, in this way, to gain one’s own identity. This interpersonal foundation of the self relies on the process of self-other differentiation. Self-other differentiation can be achieved at spatial/bodily (Noel et al. 2017) and cognitive (Sowden & Shah 2014) levels. Accordingly, self/other differentiation involves both motor processes underpinning imitation and cognitive processes linked to mentalizing and reasoning about the other’s beliefs and emotions (Eddy 2018).

In this respect, SCZ shows notable difficulties with self-other differentiation, at both body and cognitive levels. With respect to bodily/action differentiation, Liepelt et al. (2012) report that SCZ individuals have a deficit in integrating one’s own and other’s actions. Noel and colleagues (2017) report that SCZ individuals possess an extremely weak or variable bodily boundary between self and others (for the loss of the self-other boundary, see also Nelson et al. 2009; Thakkar et al. 2011; Michael and Park 2016). For example, a diminished self-other boundary distinction in SCZ is supported by a study by Delevoye-Turrell, Vienne, and Coello (2011) on the peripersonal space (PPS) representation. In this study, individuals with SCZ and controls were asked to judge the location of the boundary of PPS indicating when objects and people entered or exited their reaching space. Results revealed that individuals with SCZ were significantly more variable in their judgments, and this higher variability correlated with the Positive and Negative Syndrome Scale (PANSS) score: the more severe the symptoms, the greater variability of judgments was observed. In the view of these findings, Nasrallah (2012) even proposes to consider a new name for schizophrenia, i.e., “self-proprioception disorder.” If atypically high levels of disinhibition in the cortico-striatal networks characterize SCZ, as we have proposed, then the shattered (not-integrated) sense of self in this condition can be seen as a failure to inhibit multisensory input in the construction and maintenance of self.

In contrast to SCZ, Noel and colleagues (2017, p. 10) consider that ASD individuals possess a sharper self-other boundary than SCZ individuals, suggesting a reverse pattern of the one attested in SCZ: “SZ [i.e., SCZ] may represent a particular instance in which the distinction between self and others is exceptionally weak (i.e., too shallow of a PPS boundary), while ASD may represent a particular instance in which the distinction between self and other is exceptionally strong (i.e., too sharp of a PPS boundary).” Relating this to cross-modality, in contrast to SCZ, which is characterized by increased, uninhibited cross-modality, correlated with the self without a clear boundary, ASD is characterized by diminished, inhibited cross-modal connections, the inhibition that also extends to multisensory input necessary to fully experience and empathize with others. “In both of these cases, the experience with others is likely to be impaired, which may then cascade into socio-communicative deficits and difficulties interacting with the physical world” (p. 10). The atypical degree of empathy and the lack of clearly bounded sense of self seems especially well illustrated with the third condition we examine here, i.e., synesthesia, in particular because the essence of this condition has been attributed to enhanced cross-modality.

Mirror-touch synesthesia (MTS) is a condition associated with atypical experiences with others (Banissy and Ward 2013; de Guzman et al. 2016; Ioumpa et al. 2019). According to Banissy and Ward (2013), the amplified vicarious tactile experiences characteristic of this condition indeed depend on atypical self-other representations, namely on difficulties in the ability to differentiate representations of the self from others. This is particularly evident when individuals with MTS are administered the Enfacement Illusion test. In the Enfacement Illusion test, self-other recognition is evaluated before and after a stimulation session in which participants watch a video showing a series of images of morphed faces representing incremental changes from the participants’ faces (self) to the faces of strangers (other). They are asked to indicate when they start/stop recognizing themselves in the video for the two directions of morphing: self to other and other to self. While looking at the video, subjects also receive tactile stimulation on the cheek and simultaneously watch a similar stimulation applied to the other in the video. Tsakiris (2008) found that in typical individuals synchronous tactile stimulation while watching another person’s face being similarly touched produced a bias in recognizing one's own face: the images that the subjects had initially perceived as containing equal parts of self and other become more likely recognized as the self. Maister and colleagues (2013) developed an adapted version of this paradigm for MTS removing the tactile stimulation. The results of their study showed that subjects with MTS reported the same effect of enfacement illusion in the absence of the physical touch component, suggesting a blurring in the self-other distinction processes. As highlighted by the authors of the study, “these results suggest that observing touch on others not only elicits a conscious experience of touch in MTS, but also elicits a change in the mental representation of self, blurring self-other boundaries. This is consistent with a multisensory account of self, whereby integrated multisensory experiences maintain or update self-representations” (Maister et al. 2013, p. 802). If the representations of self depend on the precise integration of multisensory input, which in turn relies on multimodal connectivity of the brain, then it is expected that any disturbance in cross-modal connectivity may lead to an impaired sense of self. In particular, because of their enhanced cross-modality, it is expected that SCZ and synesthesia will feature a more disintegrated, more shattered sense of self, with their propensity to reach to and experience the other, while ASD will show the opposite tendencies.

The atypical multisensory processes that in SCZ, ASD, and MTS affect self-other differentiation at spatial/bodily level also have consequences for the self-other differentiation occurring at the cognitive level and underlying some competences of social cognition. As previously mentioned, both SCZ and ASD are characterized as disorders of social cognition, including of ToM. Such atypical functioning in social domains, including imitation and empathy, is also reported in MTS (de Guzman et al. 2016; Ioumpa et al. 2019). This atypical functioning of social cognition has indeed been related to atypical modulation of self-other representations not only in SCZ and ASD (Cook and Bird 2012; Ferri et al. 2012; Sowden and Shah 2014), but also in MTS (Kuang 2016). For example, deficits in ToM are typically disturbances in monitoring the intentions of others, specifically when these intentions are different from their own intentions. In this regard, it has been suggested that in SCZ the phenomenon of paranoid delusions (e.g., delusions of persecutory control) can be attributed to a deficit in managing representations of self and others, namely a deficit in the ability to construct accurate mental representations of other individuals’ intentions (Frith 1992; Allen et al. 2004; Harrington et al. 2005; Sowden & Shah 2014).

When it comes to ASD, in parallel with a sharper bodily separation between self and other, also a cognitive differentiation can be observed that is sharper than in the typical case (as opposed to being too weak, as in the case of SCZ), in the sense that ASD individuals have a difficulty viewing themselves as embedded within social contexts. In this respect, Frith & De Vignemont (2005) discuss the distinction between egocentrism and allocentrism in social cognition. For the egocentric stance, the other person is represented in relation to the self (“you”), while for the allocentric stance, there needs to exist a mental state of the other person represented as independent from the self (“he/she/they”). The egocentric perspective is a representation that comes from a direct self-knowledge; the allocentric stance is a representation of oneself as a person among others (p. 725). While neurotypical people can switch from one perspective to the other to readily achieve an integrated “sense of self,” ASD people have difficulties with switching, and they tend to take an egocentric stance.

These considerations are not only consistent with treating ASD and SCZ as diametrically opposed cognitive conditions, but more specifically, they are also consistent with ASD and SCZ exhibiting mirror patterns with respect to cross-modality, as per our proposal, given that cross-modality is above all about crossing conceptual boundaries and merging conceptual domains. Research on self-other representations in synesthesia provides further support for this view. In fact, according to Santiesteban et al. (2015), “mirror-sensory synesthesia occurs when representations of others are not properly inhibited (while representations of the self are somewhat inhibited), whereas the opposite seems to be the case with deficits in Theory of Mind (ToM) and empathy found in individuals with ASD, which occur when representations of the self are not properly inhibited and representations of others are not enhanced enough.”

## From self to language: the use of pronouns as an example

The cognitive systems underlying the construction of the self (both in neurotypical population and in SCZ, ASD, and synesthesia) are intimately tied to language: the way we use language reflects important psychological factors relating to the way we represent our own self and the self of others. Accordingly, studying some aspects of language use by people with these conditions can offer a promising window to the underlying deficits in the domains of the notion of self and, more generally, cross-modality. From a general point of view, in the light of what we have said so far about the altered sense of self in SCZ, ASD, and synesthesia, we should expect to find in these conditions atypical language features that are characterizable in terms of the diametrical oppositions. In this section, we focus on the use of pronouns, the words that typically grammaticalize the self-other distinction (and the awareness of other persons), whereas in Sect. “The self and modes of narration”, we consider the use of narratives, as one basic way of constructing one’s identity by means of autobiographical stories.

Because of their noted role in grammaticalizing the participants in any conversational exchange, a widely shared opinion is that impairments in self-other distinctions, as found in SCZ, ASD, and synesthesia, should be manifested in an atypical use of pronouns (e.g., Lombardo and Baron-Cohen 2010; Stephane et al. 2010). Children’s ability to distinguish personal pronouns “I” and “you,” or “mine” and “yours,” emerges in the second year of life (e.g., Lewis & Ramsay 2004; Moyer et al. 2015), and is considered a manifestation of self-awareness. In fact, “The correct use of pronouns requires a representation of the listener’s mind such that the speaker knows what the listener needs clarified (e.g., who exactly “she” represents in a sentence) and thus could reflect social–cognitive skills” (Buck et al. 2015). This is clearly related to ToM, as previously introduced. From this perspective, the difficulty of successfully using personal pronouns in a conversational context seems to correlate with the difficulty of recognizing the reciprocal roles of speaker and listener in conversation. According to De Villiers and De Villiers (1974), the comprehension of deictic terms “can only be understood by ‘non-egocentric’ individuals who recognize the context of the relationship between the speaker and the addressee” (Hobson 1990, p. 171). Similarly, Bruner (1975) highlights that “children need to have grasped reciprocal roles in discourse before they can understand linguistic deixis” (Hobson 1990, p. 171).

Taking all this into account, we can expect that ASD will show difficulties recognizing and maintaining the reciprocal roles in conversation, resulting in the tendency to avoid pronouns altogether (by using names instead), or to use them in atypical ways. Jordan (1989) analyzed the use of pronouns in a group of 11 children with ASD and reported that 72% of them used their own name for self-reference instead of the pronoun, while only 18% of language-matched controls did so (see also Sterponi et al. 2015, among others). As an example of such pronoun avoidance, consider having Jimmy saying “Jimmy likes apples” in lieu of “I like apples.” This is certainly attested in early child speech, and there may be a prolonged period in which this is true of children on the ASD spectrum, and even adults with ASD (Lombardo et al. 2007). It has also been shown that children with ASD tend to reverse first-person pronouns, using them to refer to other people. According to Kanner (1943, p. 244), in ASD “personal pronouns are repeated just as heard, with no change to suit the altered situation. The child, once told by his mother, “Now I will give you your milk,” expresses the desire for milk in exactly the same words.” Other investigations also reported an inappropriate use of second person pronouns. For example, it has been shown that children with ASD frequently use “You” to refer to themselves (Jordan 1989; Lee et al. 1994; Hobson et al. 2006; Shield et al. 2015). Similar atypical uses extend to third person pronouns. In a study on the use of pronouns in storytelling in people with ASD, Novogrodsky (2013) and Novogrodsky and Edelson (2016) found inappropriate uses of the third person in storytelling tasks but not in (less onerous) retelling tasks.^3^ Similarly, Hobson et al. (2010) reported that participants with ASD were less likely to use third person pronouns.^4^

Regarding SCZ, it has been suggested that the frequency of self-referential pronouns is a potential marker of psychosis (Fineberg et al. 2016; Palaniyappan 2021). In psychosis, formal thought disorder (that is, the inability to organize one’s thoughts in a logical sequence with the aim of attaining a specific goal) manifests itself in disorganized speech resulting from atypical use of many types of words, including an excessive use of pronouns (Mackinley et al. 2021; Tang et al. 2021). Rochester and Martin (1979) reported that individuals with SCZ frequently ineffectively explain to whom one is referring when using pronouns. One also finds a reduced use of first-person plural pronouns (Roy & Chiat 2013), and an increased use of first-person singular pronouns in SCZ (Buck & Penn 2015; Tang et al. 2021), which is the opposite tendency of what is found in ASD. Perhaps the preponderance of pronouns in SCZ reflects their switching of perspective too fast and too often, relative to the typical population, while ASD avoids switching perspectives, and thus also avoids pronoun use. Taken on the whole, this suggests that ASD is characterized by the avoidance of pronouns, in comparison with the typical case, while SCZ seems characterized by an excessive use of pronouns. These considerations again reveal an opposite disturbance in these two conditions, which can be related to the disturbance in the sense of self, and ultimately to the disturbance in cross-modality and brain connectivity.

When it comes to synesthesia, to the best of our knowledge, there are no studies explicitly investigating the use of pronouns in synesthetes.^5^ However, in the light of what we said in Sect. “The notion of self in SCZ, ASD and synesthesia”, we can expect to find in MTS an increased use of pronouns, particularly first-person pronouns. As mentioned above, in MTS we observe a blurring of the self-other differentiation affecting the empathic processes, which appear hyperactivated (e.g., Bolognini et al. 2013; Holle et al 2013). “Stronger empathy is expected because synesthetes experience on their own body any unpleasant sensation they observe in others: being more sensitive to other people’s misfortunes, they would be more motivated to relieve others’ suffering” (Ioumpa et al. 2019, p. 2). Accordingly, the reason why an excess of first-person pronouns can be expected for MTS (increased empathy) would contrast with the reason why first-person pronouns can be misused (or avoided) by people with ASD (increased egocentrism).

## The self and modes of narration

Another fruitful way to analyze both the construction and alteration of self in reference to language use in conditions like ASD, SCZ and synesthesia is to investigate the narrative dimension (e.g., Giddens, 1991; McAdams & McLean 2013; Allé et al. 2015; Adornetti and Ferretti 2021). This view is founded on the position that human beings construct their own identities by means of stories, e.g., by producing autobiographical narratives (Schechtman 1996; McAdams 2001; Habermas & de Silveira 2008; Altavilla et al. 2020; Canali et al. 2021). Paradigmatic of this perspective is Bruner’s constructivist model of personal identity (e.g., Bruner 2003, 2004). According to Bruner, autobiographical narratives support the integration of different aspects of personal experiences into a unitary representation. In this sense, these narratives might promote the construction of a coherently structured sense of self. In his words: “self-telling of life narratives achieves the power to structure perceptual experience, to organize memory, to segment and purpose-build the very ‘events’ of a life. In the end, we become the autobiographical narratives by which we ‘tell about’ our lives.” (Bruner 2004, p. 694). In the same vein, Dennett (1991) proposes that personal identity must be interpreted as a center of narrative gravity and Gallagher (2000) adheres to the view according to which human beings have a sense of a coherent and continuous self—i.e., a self that extends in time—because they have the ability to tell stories.

From this it transpires that certain aspects of the atypical sense of self in SCZ, ASD and synesthesia, can also be tied to (and manifest in) the disruptions of the narrative plan, which can best be illuminated by determining how the narratives generated by people with these conditions differ from those produced by neurotypical subjects. Before going into details, it is necessary to clarify what are the defining features of a narrative. At a general level, a narrative can be defined as a linguistic representation of temporally (e.g., Genette 1972) and causally (e.g., Trabasso and Sperry 1985) connected sequences of events, driven by the goals and motivations of one or more agents (Bruner 1997). Two main themes emerge from this definition: (i) the relationship between events in the flow of discourse (which boils down to the nature of the processes and the cognitive systems responsible for the causal and temporal connections between the events), and (ii) the nature of events and agency (i.e., how events and agency are represented in the mind). These are closely related themes that open the way for different strategies for analysis. We now focus on these two aspects.

### Relationship between events in the flow of discourse

Concerning the relationship between events in the flow of discourse, of relevance are both the studies analyzing the cognitive systems (e.g., executive functions and MTT: Kerns and Berenbaum 2002; Kerns 2007; Adornetti 2014; Ferretti et al. 2018; Marini et al. 2019) and those analyzing the brain structures (e.g., the hippocampus: Cohn-Sheehy et al. 2021; Milivojevic et al. 2016; Race et al. 2015), both of which are at the basis of the processing of the *global coherence* of the narrative plan. It is clear then that the systems responsible for these relationships important for discourse construction and interpretation have a cognitive foundation, and not merely a linguistic one.^6^ In this paper, we are adding the relevance of the connectivity in the cortico-striatal brain networks, which are of direct relevance for cross-modality, as previously mentioned.

That SCZ and ASD people have difficulties in managing the global coherence of a narrative is well documented (e.g., Andreasen 1979; Marini et al. 2008; Boudewyn et al. 2012; Stirling et al. 2014; Sah and Torng 2015; Boudewyn et al. 2017; Adornetti et al. 2020; Jolliffe and Baron-Cohen 2000). Specifically, it is demonstrated that some of the deficits in managing the global narrative plan affecting individuals with SCZ and ASD can be linked to difficulties in coping with the *causal connections* between the constituent events of a story (Losh and Capps 2003; Diehl et al. 2006; Allé et al. 2015; Sah and Torng 2015; Allé et al. 2016a; Willits et al. 2018). Diehl and colleagues (2006) investigated story recall and narrative coherence in children with high functioning ASD and children with typical development. Their analysis revealed that the two groups produced stories that were comparable in length and syntactic complexity, but the narratives generated by the children with ASD were significantly less coherent, i.e., with fewer causal relationships evaluated in reference to a necessity test between events (an event A was considered to be necessary for an event B in that if event A has not occurred, event B would not have occurred). Analogous results have been found also where the causal relationships were evaluated in reference to explicit linguistic causal connections, such as “because,” “that’s why,” “so” (King et al. 2014). A similar pattern is also evident in the narratives produced by people with SCZ. Willits and colleagues (2018) examined the semantic cohesion of personal narratives in SCZ and found that they contained few connectives (e.g., *because*, *and*, *although*) to link words and phrases within sentences. According to the authors, “this means their audience was left without important cohesive links to connect ideas within a sentence and had to devote cognitive resources to make these connections” (p., 368). Difficulties of this kind in SCZ can be observed also in narrative comprehension. Examining the modulation of the N400^7^ in a discourse context, Ditman and Kuperberg (2007) revealed that SCZ individuals have difficulties constructing coherent links across individual sentences to build up overall global context in discourse comprehension.

Some of the difficulties in the processing of the global narrative plan affecting individuals with SCZ and ASD have also been referred to as problems in representing the *temporal connections* between events in the flow of discourse (Allé et al. 2015; Allé et al. 2016a, b; Ferretti et al. 2018; Marini et al. 2019). At a general level, several lines of investigation suggest that a crucial role in this regard is played by the hippocampus (e.g., Duff et al. 2008; Race et al. 2015; Cohn-Sheehy et al. 2021), a brain structure embedded in the temporal lobe, which has been proposed to be one of the main neural substrates of MTT (e.g., Maguire et al. 2016). In a study on amnesic individuals with medial temporal lobe damage, Race and colleagues (2015) found that hippocampus is involved in “the integration of linguistic elements into cohesive and coherent discourse units when constructing several different types of complex verbal narratives” (p., 279). The authors highlighted that these individuals had difficulties both in the production of fictional narratives and in the generation of personal stories about future and past events.

The findings by Race et al (2015) are particularly relevant in interpreting some peculiarities of the narrative deficits affecting individuals with SCZ and ASD, given that reductions in the neural circuits typically associated with MTT (e.g., in the hippocampus) have been observed in these conditions (e.g., Dager et al. 2007; Zhou et al. 2008; Fornara et al. 2017; Cachia et al. 2020; Banker et al. 2021). In line with these findings, Ferretti and colleagues (2018) showed that children with ASD with impaired MTT exhibited also impairments in generating coherent fictional stories. In this study, children with ASD were asked to generate past and future episodes of a narrative discourse and to complete a task aimed at assessing one of the subcomponents of MTT: their ability to project into a future scenario. Their performance was compared to that of a group of children with typical development. The results showed that children with ASD who were impaired in the ability to project into the future also performed significantly worse on the narrative production task: their fictional narratives contained more errors of global coherence (i.e., elements that were tangential or incongruent with the temporal storyline), more repetitions, and fewer causal links than the narratives of the children with typical development (see also Marini et al. 2019). These kinds of deficits also extend to the personal narratives of individuals with ASD (e.g., McCabe et al. 2013). Similarly, persons with SCZ show difficulties in managing the temporal dimension of a narrative. It has been shown that when people with SCZ are asked to narrate their life stories, they have difficulties to organize and locate events chronologically across the course of the narrative (Allè et al. 2015; Allé and colleagues 2016a, b). Analyzing the temporal macrostructure of their life stories (i.e., elaboration of beginnings and endings, number of temporal indicators, number of anachronies in the narrative), Allé and colleagues (2016b) found that the global temporal coherence is significantly reduced in individuals with SCZ and that “the lower temporal coherence in patients seems mainly associated with their tendency to spontaneously deviate from the chronological order of events without marking the deviation” (p. 26). As is the case with ASD, also in SCZ the difficulties in managing the global temporal coherence seem to be related to anomalous functions of MTT. Research has indeed shown that individuals with SCZ also have reduced ability to retrieve both specific events from their personal past and to imagine possible scenarios that might happen in their personal future (e.g., Cuervo-Lombard et al. 2007; D'Argembeau et al. 2008; Raffard et al. 2013; Chen et al. 2016; Lyons et al. 2016; Yang et al. 2018; Malek et al. 2019).

From what has been said so far, it can be concluded that part of the difficulties in the construction of self in individuals with SCZ and ASD can be linked to analogous difficulties in managing the narrative global coherence (the property that governs the causal and temporal relationship between events). Interestingly, when a narrative is analyzed in reference to the temporal and causal connections between the events, more similarities rather than differences between ASD and SCZ seem to emerge. These similarities might be explained in reference to an anomalous activity of the hippocampus in ASD and SCZ affecting the binding process between events. Indeed, it has been proposed that the hippocampus supports the integration of separated events into larger narratives (e.g., Cohn-Sheehy et al. 2021; MacKay et al. 1998, 2007; Race et al. 2015). On the other hand, as shown in the next section, these conditions do exhibit opposite patterns when considering the second aspect involved in the definition of a narrative: the nature of event representations and agency.

### Event representation and agency

Although the notion of event has a long philosophical tradition, our focus here is on event representation and agency (i.e., how they are represented in the mind). Following Sinha and Gärdenfors (2014), events often call into play situations “in which selves and other people figure as agents, performing actions directed to other agents and to objects” (2014, p. 1). More specifically, referring to the theory of conceptual spaces (Gärdenfors 2000, 2014), the two authors describe such events “as built up from an agent, an action, a patient, and a result (…). The result of an event is modeled as a change vector representing the change of properties before and after the event” (Sinha and Gärdenfors 2014, p. 5). According to Sinha and Gärdenfors, there is a very strong relationship between events and language (here referring to the propositional structure of utterances), given that “event structure, the combination of constituents encoding objects, actions, location, and motion, is the fundamental building block for sentence meaning and grammar” (Sinha and Gärdenfors 2014, p. 1). Challenging this latter view, Radwansky and Zacks (2014) present a different perspective—The Event Horizon Model—according to which the representation of events is involved in discourse processing, rather than (or more than) in sentence processing. In their opinion, discourse processing is driven by generically cognitive rather than specifically linguistic systems: understanding discourses implies processes such as the ability to distinguish one event from another (*event segmentation*), also involved in the segmentation of the continuous flow of events in everyday life. Furthermore, the segmentation of events calls into play prospective forms of cognition. The anticipatory and predictive character of the segmentation of events plays a leading role in narrative competence, given that “one major function of event models in language comprehension is to enable predictions about what information is coming in a discourse” (Radwansky and Zacks 2014, p. 59). Experimental investigations prove the importance of event segmentation in narrative comprehension. For example, Zacks et al. (2009) showed that, when reading a narrative, clauses relating to the representation of the transition points between one event and another (event boundaries) are read more slowly (i.e., required a greater effort on the part of the reader) than the clauses relating to the representation of an event. An increase in processing time coinciding with the segmentation of events during reading has also been observed in experiments with neuroimaging techniques (Speer et al. 2007).

When the narrative competence of SCZ and ASD is investigated in reference to the crucial role of the representation of events, an opposite phenotype between the two conditions emerges. In people with ASD, the atypical representation of events can be ascribed to an *anchoring deficit*, which results in a super-focus on the details constituting an event. As pointed out above (following Benítez-Burraco and Progovac 2021), ASD is characterized by impoverished global connectivity in the cortico-striatal networks, but also by enhanced local connectivity, which is responsible for their sharper attention to detail. This general characterization can also help explain the bias in their narratives, leading individuals with ASD to process information locally focusing on details, while failing to integrate them into a wider global context (Happé et al. 2001)—details of events seem to exert a strong attractive power that prevents ASD from passing from the particular to the global scene of the story. This *anchoring deficit* might be brought into play to account for alterations in the use of anaphoric pronouns often observed in ASD (e.g., Colle et al. 2008).

Given this view, with ASD narratives one can expect to find super-focus, associated with a heightened attention to detail, perhaps with some (compulsive) repetitions. In support of this, Jolliffe and Baron-Cohen (2000) showed that the tendency to focus on details makes it difficult for people with ASD to integrate the constituent events of a story into a higher-order sequence: anchored to the details of the scenario, subjects with ASD struggle to connect one event to another and, therefore, to build the sequence of events into a story (see also Nuske and Bavin 2011; Adornetti et al. 2020). As also pointed out in Ferretti and colleagues (2018), although the causes of narrative problems in ASD are probably multiple, one of them may be a lack of imagination. Results of this study revealed that children with ASD who were asked to imagine the possible future or past episodes with respect to the target stimuli tended to persist on describing the elements included in the pictures’ stimuli (*super-focus*) rather than introducing novel events connected to the pictures’ stimuli, as the children of the control group did. Similarly, Marini and et al. (2020) found that children with ASD who were administered a narrative description task produced fewer ideas than those depicted in the vignettes, suggesting a “significant difficulty … in the phase of nonverbal conceptualization of the story” (p., 9). Overall, these findings are consistent with the proposal that ASD is characterized by an impairment, more precisely an inhibition, in the global connectivity of the relevant brain networks involved in cross-modality, while it shows a strength in local connectivity; both of these characteristics can be seen as yielding narratives which are super-focused on the details of events, but lacking in global cohesion.

In our view, SCZ presents with an opposite pattern, which follows from a different way of representing events and agency, i.e., from atypical sequencing of complex dynamic events (e.g., Zalla et al. 2004, 2006), which has repercussions on the narrative plan. In contrast to what happens in ASD, in SCZ we observe narratives with an unfocused, scattered, wildly metaphorical and imaginative style. The speech of SCZ individuals is often described as disorganized and lacking focus (e.g., Kuperberg 2010); as exhibiting “loose associations” (Chaika 1974; Nestor et al. 1998), and novel metaphors and novel words, including gibberish and neologisms (Chaika 1974). All these symptoms may indeed have to do with uninhibited, enhanced cross-modality, as highlighted by Surguladze et al. (2002): in SCZ “increased cross-modal connectivity (reduced modality modularity and informational encapsulation) between lexical representations … could result in impaired language, particularly speech processing” (p. 884). For example, “loose associations” have to do with jumping from topic to topic, in such a way that to a typical language user seems incoherent, i.e., lacking in focus and sense (e.g., Kuperberg 2010; Ankofski 2018). Interestingly, sometimes the loose associations come from paying special attention to the meaning of a word from the previous utterance, rather than focusing on the overall topic (see, e.g., Chaika 1974).^8^ In this respect, SCZ can be seen as using super metaphorical language, with connections not obvious or acceptable to, and thus interpreted as “word salad” or “gibberish” by a typical language user. Another interesting case in which the speech of persons with SCZ is characterized by connections not obvious to others is when it relies on forms of *clanging*: “a pattern of speech in which sounds rather than meaningful relationships appear to govern word choice … and redundant words are introduced. In addition to rhyming relationships, this pattern of speech may also include punning associations, so that a word similar in sound brings in a new thought” (McKenna and Oh 2005, p. 23). Relevant for us, these forms of clanging echo typical synesthetic mechanisms. Nonetheless, the way individuals with SCZ are using their language seems to be on a continuum with what metaphorical language generally affords, especially if we also consider poetry (see footnote 8).

So far, we have analyzed the nature of the narrative self in ASD and SCZ in terms of a diametrical opposition between them, relating the impairments in both to atypical cross-modality. Considering that synesthesia is a condition characterized by super cross-modality (e.g., Cuskley and Kirby 2013), the question arises as to what we can say about narrative abilities of synesthetes in the context of self. In the previous sections, we examined how different levels of self-awareness (senses of ownership and agency, self-other distinctions) can be impaired in certain kinds of synesthesia. While we can expect that a similar pattern of atypical narrative construction of the self will be observed in synesthesia, to the best of our knowledge, there are no studies investigating the narrative abilities, including the life stories, of synesthetes. That said, given that cross-modality seems to be directly involved in the representation of events, it is legitimate to hypothesize that an atypically enhanced cross-modality, such as that observed in synesthesia in general, might affect the narrative level in this respect, falling somewhere between ASD and SCZ.

## Discussion and conclusions

In this paper, we have proposed that many of the issues with discourse experienced by people with SCZ and ASD, and to some extent synesthesia as well, are related to problems with event representation in narratives, which in turn boil down to an altered sense of self. The novelty of our approach is in attributing the altered sense of self in all three conditions to the mechanism of cross-modality, which relies on a precise balance between inhibition and disinhibition in the connectivity in selected cortico-striatal brain networks, which also govern the suppression of (reactive) aggression in humans, and are responsible for syntactic and linguistic processing more generally, among several other functions. We further argued that this is the reason why all these dimensions cluster together in cognitive conditions like SCZ, ASD, and to some extent synesthesia, and also why these conditions often present as different points (diametrically opposed in the case of SCZ and ASD) within the continuum of the social brain, when it comes to: aggression management, metaphorical thinking, and language patterns, which are core features characterizing the cognitive and behavioral phenotype of the human species. These conclusions build on previous research by Benítez-Burraco and colleagues (Benítez-Burraco and Progovac 2021; Benítez-Burraco et al. 2021), where they argued that a common neuronal substrate (i.e., enhanced connectivity in the cortico-striatal networks), and a common evolutionary cause (i.e., a gene-culture feedback loop, implicating HSD and the emergence of early grammars, e.g., Progovac and Benítez-Burraco 2019) can be invoked to unify core aspects of language evolution that are usually treated independently: cross-modality, (verbal) aggression, structural language processing, and modern uses of language (including discourse abilities as found in present-day communication). In this paper, we have extended this paradigm to other cognitive and behavioral dimensions, particularly, the notion of the self and discourse practices.

With regard to ASD, the link between cross-modality and self-construction can be observed at different levels of analysis. In this paper, we considered the effects that atypical inhibition of cross-modality found in ASD has on the basic aspects of self, such as ownership, agency, and self-other differentiation. The reduced cross-modality characterizing ASD also has effects on the relationship between self and narratives. As we have seen, the representation of events in ASD appears impaired especially when it comes to the construction of a coherent sequence of events characterizing the global level of a story. Altered narratives of this kind by people with ASD can be linked to an atypical sense of self, which can in turn be related to diminished cross-modality and brain connectivity in cortico-striatal networks, but also associated with the diminished ability for MTT.

With regard to SCZ, the issues involving the fractured sense of self and atypical narrative structures in the stories created by people with this condition can be analyzed as related to their super cross-modality, attributed to the abnormally enhanced, disinhibited connectivity in the cortico-subcortical brain networks. This enhanced connectivity would lead not only to “loose associations” in speech and narratives, and to the excessive, somewhat chaotic use of pronouns, but also to hallucinations, i.e., surreal experiences (Silbersweig et al. 1995), which in turn lead to the surreal sense of who controls the actions, and thus to the shattered sense of self. In this way, our approach can unify the linguistic with non-linguistic symptoms of SCZ, providing a common cause.

Synesthesia can shed further light on these phenomena, given that it is characterized as a condition exhibiting super cross-modality. According to Cuskley and Kirby (2013: 3), “synesthetes should be viewed as super cross-modal associators, with unusually strong, stable, and specific cross-modal biases.” Importantly, the enhanced cross-modality in synesthesia has been ascribed to the hyper-connectivity of a synesthete’s brain (Hänggi et al. 2011; Jäncke et al. 2009; Rouw et al. 2011; O’Dowd et al. 2019; Van Leeuwen et al. 2020). This is consistent with the observed parallels between synesthesia and SCZ, including vivid imagination which characterizes both, even though with SCZ such imagination seems more severely blurred with reality. In this sense, various kinds of hallucinations experienced by individuals with SCZ may be considered as more heightened, pathological states of synesthesia, as proposed in Benítez-Burraco and Progovac (2021).^9^ Conversely, as noted by Rothen and Meier (2013), the more vivid imagery visualizer style in synesthesia is consistent with reports of hallucinations and fantasizing (see also Banissy et al. 2012; 2013), and difficulties in dissociating dream imagery from reality (Rothen and Meier 2013, 3). In fact, Banissy and colleagues (2012) found that synesthetes score higher on positive schizotypy.

The complicating factor regarding synesthesia is that there are many different types, showing hyper-connectivity across different brain regions. The primary mechanism for grapheme-color synesthesia, one of the most common types, has been reported to involve cortico-cortical interactions (e.g., Rouw & Scholte 2007). Nonetheless, subcortical structures seem to be implicated, too: according to, e.g., Ramachandran and Hubbard (2001), hyper-connectivity between the sensory cortex and amygdala in grapheme-color synesthesia would explain the heightened aversion synesthetes experience when seeing numbers printed in the “wrong” color. Cytowic (1993) also emphasizes the involvement of the limbic system in synesthesia.

Another relevant type of synesthesia is misophonia, a neurological condition in which negative experiences are triggered by specific sounds. Edelstein and colleagues (2013) hypothesized that a distortion of connections between the auditory cortex and limbic structures causes this kind of sound-emotion synesthesia. Interestingly, Daniels, Rodriguez, and Zabelina (2020) report higher anxiety levels at baseline with this group, as well as diminished cognitive control, with a significant percentage of individuals resorting to verbal and/or physical aggression in response to triggers (Schröder et al. 2013). Schröder et al. (2019) report “intense rage and disgust” provoked by hearing specific human sounds in misophonia. As there is still much to learn about the exact mechanisms underlying various types of synesthesia, we leave it for future research to determine the extent to which subcortical structures may be involved in each of these types.

As mentioned in Sect. “The notion of self in SCZ, ASD and synesthesia”, synesthesia also sometimes presents with a disturbance in the sense of self. In order to highlight the link with cross-modality (and with the mechanism it relies on, i.e., enhanced brain connectivity), it is important to point out, as discussed mostly in Sect. “The notion of self in SCZ, ASD and synesthesia”, that the typical construction and maintenance of self relies on a multimodal/multisensory foundation, which is the essence of cross-modality. In the typical case, there seems to exist a delicate balance between maintaining a separate, integrated notion of self, and extending one’s self (in space and possibly time) to empathize with others, with these two different perspectives shifting with different contexts. In the atypical cases, as we propose, this balance is disturbed, in two diametrically opposed ways, which, moreover, can be linked to the disturbances in the mechanism of cross-modality.

On the one hand, super cross-modality can be seen as supporting enhanced empathy through an atypical extension of the self to others (a shattered sense of self, as found in SCZ and some forms of synesthesia), but less ability to exit that perspective and solidify one’s own sense of self as separate from others. This also seems to correlate with the excessive and chaotic use of pronouns in SCZ, as discussed in Sect. “From self to language: the use of pronouns as an example”. After all, the use of pronouns (I, you, she) is variable, and it is all about the relationships between self and others, while names (John, Maria) are more constant, more independent, and do not vary as much across contexts and perspectives. On the other hand, an atypical case of diminished, inhibited cross-modality, as attributed to ASD, shows the opposite effects on the construction and maintenance of self. In ASD, the sense of one’s own self is more independent, and more separate from others, than in the typical case, which can be seen as correlated with a diminished degree of extension of one’s self to others, and thus of empathy with others. This also can shed light on the opposite trend in the use of pronouns: while pronouns are used excessively in SCZ, they are often avoided in ASD, with, e.g., the first-person pronoun “I” often being replaced by the name of the speaker. As pointed out above, names can stand independently and separately from others, while pronouns are constantly in a flux, and always in relation to another.

From what has been discussed in Sect. “The self and modes of narration”, it follows that the two processes characterizing the construction of the narrative plan—the relationship between events in the flow of discourse and event representation and agency—are both involved in the construction of the narrative identity and, consequently, in the explanation of some of the disturbances of the self in SCZ and ASD, as characterized above. While clear differences do not seem to emerge between the two conditions when it comes to the former factor, i.e., the relationship between events, it is in reference to the latter factor, i.e., the representation of events and agency, that the view of ASD and SCZ as diametrically opposed conditions can be maintained. Specifically, our proposal is that the different ways of representing events in ASD and SCZ (and arguably in synesthesia as well) can be interpreted in reference to the atypical inhibition vs. disinhibition of the mechanism of cross-modality, necessary, among many other different functions, to support metaphorical extensions in language.

In other words, our proposal is that atypical features of narratives by people with SCZ and ASD also result from altered cross-modality, and ultimately from altered connectivity in the cortico-striatal brain networks. As pointed out above, the impairment in narrative structure is also related to the altered sense of self, which in turn receives an explanation in the realm of cross-modality. Eventually, we expect that all these alterations result, at least in part, from an abnormal presentation of HSD features in these conditions. As shown in previous research on SCZ (Benítez-Burraco et al. 2017) and ASD (Benítez-Burraco et al. 2016), candidates for these conditions are enriched in genes involved in HSD. As HSD during human evolution contributed to an enhanced cross-modality via reduced reactive aggression (Benítez-Burraco and Progovac 2021), our proposal here is that these evolutionary events in turn brought about a more modern representation of the self and more modern discourse forms and practices. Our hypothesis is thus that the mutation of selected genes that are candidates for SCZ or ASD, but that are also involved in HSD, resulted in SCZ or ASD symptoms via the deleterious effect of these mutations on the management of aggression and cross-modality, bringing about, specifically, an altered notion of self and problems with discourse more generally, as discussed in the paper.

At the same time, our proposal is not incompatible with some recent proposals relating MTT abilities to the hippocampus. For example, Corballis (2019) proposed that the evolutionary changes in the hippocampus contributed to an enhancement of our episodic memory, which allows us to mental-travel both forward and backward in time, which in turn refined our creative imagination. As we argued in the previous sections, problems with mental travel, as well as with imagination, can be partly responsible for the atypical narrative structure in people with ASD and SCZ. Complementary to Corballis’ proposal, Benítez-Burraco (2021) has proposed to relate these evolutionary changes in the hippocampus to HSD, via the changes in the hippocampal function and size, which are associated with the changes in stress management, as features of HSD increased. In animals, including humans, the hippocampus is primarily involved in constructing spatial maps (Maguire et al. 1998; Vinogradova 2001). In this sense, changes in the hippocampus and increased cross-modality, i.e., enhanced connectivity in the cortico-subcortical brain circuits), both of which are linked to HSD, would have enabled humans to build upon this more primitive ability for spatial mental travel by (metaphorically) extending it to mental time travel.

How exactly the hippocampus can be integrated in this network is a question for future research to address. In our view, it is presently not possible to draw a clear distinction between the mechanisms behind cross-modality and the mechanisms behind MTT, in the construction of self in narratives: rather than being separate functions, they probably work closely together in the narrative processing underlying the construction of the narrative self. In fact, MTT can be seen as overlapping with cross-modality, particularly, when it comes to metaphoricity. This is so because the expression of time in natural languages, as well as conceptualization of time, is largely a metaphorical extension of space. In this respect, humans typically (but not always, as cultures can differ in this respect) perceive time as being linear, organized neatly on a line that has a dot for the Present moment, and then one treats events to the left of that dot as Past, and those to the right of that dot as Future. Languages routinely use metaphors of space to express time, in expressions such as “This issue is now behind us./It is no use looking back.” Or “Better times are ahead of us.” Prepositions that express movement toward something can be grammaticalized as future markers, e.g., English “to” is used spatially in “He walked to the store” but temporally in infinitives that imply future, such as “The president to visit China.” In English one also talks about “long meetings,” clearly using a spatial adjective, as found in, e.g., “long lines.” In addition, English “going to” is another grammaticalized metaphorical expression denoting future time, indicating both movement and the direction toward something, as evident in the contrast between its spatial use in “I am going to the store” and its temporal use in “I am going to faint” (for many more examples and patterns of metaphoricity, and its central role in language, see Lakoff and Johnson 1980; for cultural aspects and differences in this regard, see Everett 2013).^10^

Accordingly, mental time maps can be regarded, to some extent, as metaphorical extensions of mental spatial maps. As a consequence, the problems that people with SCZ and ASD experience with MTT can partly have to do with MTT needing to rely on metaphorical extension, which in turn relates to cross-modality and the connectivity of the brain. As pointed out in Sect. “Introduction”, ASD individuals have a general problem with metaphorical extension, due to, as we have argued, more limited access to the connectivity in the brain networks that support cross-modality. On the other hand, the opposite seems to hold for SCZ, where an enhanced degree of disinhibition in these cortico-striatal networks seems to lead to unconstrained cross-modality, and with it to over-metaphorizing, and ultimately hallucinations and delusions. Last, but not least, the fact that HSD impacted on both hippocampal function and cross-modality provides additional support for the view of an interplay between cross-modality and MTT, and ultimately, between selected cortico-striatal circuits and the hippocampus. This issue certainly deserves further attention and elaboration, and we leave it for future research.

## Data Availability

The materials used in the paper are presented as a list of bibliographical references at the end of the paper.

## Footnotes

1 In this respect, ASD shows an aptitude for rule-based systems and for hyper-systemizing (e.g. Baron-Cohen 2009; Ward et al. 2017), which also contrasts with SCZ.

2 Weak central coherence and field-dependence/field independence are two clearly related constructs, as proven by the fact that often the same tasks -i.e., the Embedded Figures Test – are used to measure them (Milne and Szczerbinski 2009). However, the exact nature of their relationship is a matter of debate. According to Happé and Frith (2006, p. 16), “It might be a mistake … to equate FI [field-independence] with weak central coherence; while FI people are conceptualised as succeeding on EFT [i.e., Embedded Figures Test] because of their ability to see, but resist, the gestalt, people with weak coherence are postulated to be good at this test precisely because they do not spontaneously attend to the gestalt, instead seeing the figure first in terms of its parts”.

3 Interestingly, this inappropriate use of third person pronouns was not limited to specific syntactic structure, which to the authors suggested “a cognitive deficit in monitoring the listener’s mental model” (Novogrodsky and Edelson 2016, p. 250).

4 However, according to Finnegan et al. (2021), although pronominal reversal was once considered a defining characteristic of peculiar speech in ASD (APA, 1980), pronoun difficulties in individuals with ASD may be more nuanced than previously imagined; on this point, see also: Barokova and Tager-Flusberg (2020).

5 Interestingly for us, a study conducted by Pierce (2018) suggests a correlation between synesthesia and transgender identity, which is characterized by context-specific use of both roles and pronouns (Webster 2019).

6 Radwansky and Zacks (2014) write: “although causal information is conveyed in a text via the words used, causal relationship information appears to be primarily represented at the event model level, not the surface or text base levels (…). Causal relations serve as the backbone for understanding and remembering the narrative as a whole” (Radwansky and Zacks, 2014, p. 76).

7 The N400 is a component of the human scalp-recorded event-related brain potential (ERP) that reflects the semantic integration of words into a preceding context (word, sentence, or discourse) (e.g., Kutas and Hillyard 1980; Kutas and Federmeier 2011; Nieuwland and van Berkum 2006).

8 As one illustration, consider one SCZ person’s response to the question of what her mother’s name was: My mother’s name is Bill…and coo? St. Valentine’s Day is the startin’ of breedin’ season of birds. All buzzards can coo. I like to see it pronounced buzzard rightly. They work hard. So do parakeets. Responding with the name “Bill” causes the person to spontaneously utter the idiom, “bill and coo”, which has romantic connotations. This seemingly takes the individual to mention Valentine’s Day, a holiday centered around romance. On closer inspection, this string of sentences would probably not be considered unusual at all if found in a book of poetry.

9 For other proposals and views regarding both overlaps and distinctions between synesthesia and SCZ, see e.g. Van Leeuwen et al. (2020).

10 For metaphorical extensions in grammaticalization processes more generally, the reader is referred to e.g. Hopper and Traugott (2003); Heine and Kuteva (2007); Smith and Höfler (2014).

## References

[CR1] Abu-Akel A (2003). A neurobiological mapping of theory of mind. Brain Res Rev.

[CR2] Adornetti I, Ferretti F, Cardella V, Gangemi A (2021). The narrative self in schizophrenia and its cognitive underpinnings. Psychopathology and philosophy of mind. What mental disorders can tell us about our minds.

[CR3] Adornetti I, Chiera A, Deriu V, Altavilla D, Lucentini S, Marini A, Vicari S, Ferretti F (2020). An investigation of visual narrative comprehension in children with autism spectrum disorders. Cogn Process.

[CR4] Adornetti I (2014) A neuro-cognitive perspective on the production and comprehension of discourse coherence. In: Chruszczewski P, Rickford J, Buczek K, Knapik A, Mianowski J, Wacewicz S, Żywiczyński P (eds), Ways to Protolanguage 3, Wydawnictwo WSF, Wrlowlac, pp. 9–24

[CR5] Aimola-Davies AM, White RC (2013). A sensational illusion: vision-touch synaesthesia and the rubber hand paradigm. Cortex.

[CR6] Allé MC, Potheegadoo J, Köber C, Schneider P, Coutelle R, Habermas T, Danion JM, Berna F (2015). Impaired coherence of life narratives of patients with schizophrenia. Sci Rep.

[CR7] Allé MC, Gandolphe MC, Doba K, Köber C, Potheegadoo J, Coutelle R, Habermas T, Nandrino JL, Danion JM, Berna F (2016). Grasping the mechanisms of narratives' incoherence in schizophrenia: an analysis of the temporal structure of patients' life story. Compr Psychiatry.

[CR8] Allé MC, d’ Argembeau A, Schneider P, Potheegadoo J, Coutelle R, Danion JM, Berna F (2016). Self-continuity across time in schizophrenia: an exploration of phenomenological and narrative continuity in the past and future. Compr Psychiatry.

[CR9] Allen PP, Johns LC, Fu CH, Broome MR, Vythelingum GN, McGuire PK (2004). Misattribution of external speech in patients with hallucinations and delusions. Schizophr Res.

[CR10] Altavilla D, Acciai A, Deriu V, Chiera A, Adornetti I, Ferretti F, Bassi A, Colonna Z, de Luca V, Canali S (2020). Linguistic analysis of self-narratives of patients with gambling disorder. Addict Disord Treat.

[CR11] Andreasen NC (1979). Thought, language and communication disorders: II diagnostic significance. Arch Gen Psychiatry.

[CR12] Ankofski D (2018). Semantic processing in schizophrenia: the effects schizophrenia has on the extended wernicke’s area.

[CR13] Arnaud S (2020). Self-consciousness in autism: a third-person perspective on the self. Mind Lang.

[CR14] Asperger H (1944). Die autistischen Psychopathen im Kindesalter. Archiv Fur Psychiatrie Und Nervenkrankheiten.

[CR15] Bailey A, Phillips W, Rutter M (1996). Autism: towards an integration of clinical, genetic, neuropsychological, and neurobiological perspectives. J Child Psychol Psychiatry.

[CR16] Bakhshi K, Chance SA (2015). The neuropathology of schizophrenia: a selective review of past studies and emerging themes in brain structure and cytoarchitecture. Neuroscience.

[CR17] Baldwin JM (1902). Social and ethical interpretations in mental development.

[CR18] Banissy MJ, Ward J (2013). Mechanisms of self-other representations and vicarious experiences of touch in mirror-touch synesthesia. Front Hum Neurosci.

[CR19] Banissy MJ, Kadosh RC, Maus GW, Walsh V, Ward J (2009). Prevalence, characteristics and a neurocognitive model of mirror-touch synaesthesia. Exp Brain Res.

[CR20] Banissy MJ, Cassell JE, Fitzpatrick S, Ward J, Walsh VX, Muggleton NG (2012). Increased positive and disorganised schizotypy in synaesthetes who experience colour from letters and tones. Cortex.

[CR21] Banker SM, Gu X, Schiller D, Foss-Feig JH (2021). Hippocampal contributions to social and cognitive deficits in autism spectrum disorder. Trends Neurosci.

[CR22] Barokova M, Tager-Flusberg H (2020). Person-reference in autism spectrum disorder: developmental trends and the role of linguistic input. Autism Res.

[CR23] Baron-Cohen S (2009). Autism: the empathizing-systemizing (ES) theory. Ann N Y Acad Sci.

[CR24] Baron-Cohen S, Harrison J, Goldstein LH, Wyke M (1993). Coloured speech perception: is synaesthesia what happens when modularity breaks down?. Perception.

[CR25] Benítez-Burraco A (2021). Mental time travel, language evolution, and human self-domestication. Cogn Process.

[CR26] Benítez-Burraco A, Progovac L (2020). A four-stage model for language evolution under the effects of human self-domestication. Lang Commun.

[CR27] Benítez-Burraco A, Progovac L (2021). Language evolution: examining the link between cross-modality and aggression through the lens of disorders. Philos Trans R Soc B.

[CR28] Benítez-Burraco A, Lattanzi W, Murphy E (2016). Language impairments in ASD resulting from a failed domestication of the human brain. Front Neurosci.

[CR29] Benítez-Burraco A, Di Pietro L, Barba M, Lattanzi W (2017). Schizophrenia and human self-domestication: an evolutionary linguistics approach. Brain Behav Evol.

[CR30] Benítez-Burraco A, Ferretti F, Progovac L (2021). Human self-domestication, pragmatics, and language evolution. Cognit Sci.

[CR31] Blakemore SJ, Bristow D, Bird G, Frith C, Ward J (2005). Somatosensory activations during the observation of touch and a case of vision–touch synaesthesia. Brain.

[CR32] Bleuler E (1911) Dementia praecox or the group of schizophrenias. Translated by J Zinkin. International Universities Press, New York, NY, 1950

[CR33] Bo S, Forth A, Kongerslev M, Haahr UH, Pedersen L, Simonsen E (2013). Subtypes of aggression in patients with schizophrenia: the role of psychopathy. J Forensic Psychiat Psychol.

[CR34] Bolognini N, Miniussi C, Gallo S, Vallar G (2013). Induction of mirror-touch synaesthesia by increasing somatosensory cortical excitability. Curr Biol.

[CR35] Botvinick M, Cohen J (1998). Rubber hands ‘feel ’touch that eyes see. Nature.

[CR36] Boudewyn MA, Carter CS, Swaab TY (2012). Cognitive control and discourse comprehension in schizophrenia. Schizophr Res Treat.

[CR37] Boudewyn MA, Carter CS, Long DL, Traxler MJ, Lesh TA, Mangun GR, Swaab TY (2017). Language context processing deficits in schizophrenia: the role of attentional engagement. Neuropsychologia.

[CR38] Bruner J (1975). From communication to language. A psychological perspective. Cognition.

[CR39] Bruner J (1997). A narrative model of self-construction. Ann N Y Acad Sci.

[CR40] Bruner J (2003). Self-making narratives. Autobiographical memory and the construction of a narrative self.

[CR41] Bruner J, Angus L, McLeod J (2004). The narrative creation of self. The handbook of narrative and psychotherapy: Practice, theory, and research.

[CR42] Buck B, Penn DL (2015). 2015 Lexical characteristics of emotional narratives in schizophrenia: relationships with symptoms, functioning, and social cognition. J Nerv Ment Dis.

[CR43] Buck B, Minor KS, Lysaker PH (2015). Differential lexical correlates of social cognition and metacognition in schizophrenia; a study of spontaneously-generated life narratives. Compr Psychiatry.

[CR44] Cachia A, Cury C, Brunelin J, Plaze M, Delmaire C, Oppenheim C, Medjkane F, Thomas P, Jardri R (2020). Deviations in early hippocampus development contribute to visual hallucinations in schizophrenia. Transl Psychiatry.

[CR45] Canali S, Altavilla D, Acciai A, Deriu V, Chiera A, Adornetti I, Bassi A, Colonna Z, de Luca V, Vignola F, Ferretti F (2021). The narrative of persons with gambling problems and substance use: a multidimensional analysis of the language of addiction. J Gambl.

[CR46] Cannon TD (2015). How schizophrenia develops: cognitive and brain mechanisms underlying onset of psychosis. Trends Cogn Sci.

[CR500] Cascio CJ, Foss-Feig JH, Burnette CP, Heacock JL, Cosby AA (2012). The rubber hand illusion in children with autism spectrum disorders: delayed influence of combined tactile and visual input on proprioception. Autism.

[CR47] Chaika E (1974). A linguist looks at “schizophrenic” language. Brain Lang.

[CR48] Chen XJ, Liu LL, Cui JF, Wang Y, Chen AT, Li FH, Wang WH, Zheng HF, Gan MY, Li CQ, Shum DH (2016). Schizophrenia spectrum disorders show reduced specificity and less positive events in mental time travel. Front Psychol.

[CR49] Cioffi MC, Banissy MJ, Moore JW (2016). ‘Am I moving?’An illusion of agency and ownership in mirror-touch synaesthesia. Cognition.

[CR50] Cohn-Sheehy BI, Delarazan AI, Reagh ZM, Crivelli-Decker JE, Kim K, Barnett AJ, Zacks JM, Ranganath C (2021). The hippocampus constructs narrative memories across distant events. Curr Biol.

[CR51] Colle L, Baron-Cohen S, Wheelwright S, Van Der Lely HK (2008). Narrative discourse in adults with high-functioning autism or Asperger syndrome. J Autism Dev Disord.

[CR52] Cook JL, Bird G (2012). Atypical social modulation of imitation in autism spectrum conditions. J Autism Dev Disord.

[CR53] Cooley CH (1902). Human nature and the social order.

[CR54] Corballis MC (2019). Mental time travel, language, and evolution. Neuropsychologia.

[CR55] Cosentino E (2011). Self in time and language. Conscious Cogn.

[CR56] Crespi BJ, Hughes D, Dettorre P (2008). Language unbound: genomic conflict and psychosis in the origin of modern humans. Social communication.

[CR57] Crespi B, Badcock C (2008). Psychosis and autism as diametrical disorders of the social brain. Behav Brain Sci.

[CR58] Cuervo-Lombard C, Jovenin N, Hedelin GUY, Rizzo-Peter L, Conway MA, Danion JM (2007). Autobiographical memory of adolescence and early adulthood events: an investigation in schizophrenia. J Int Neuropsychol Soc.

[CR59] Cuskley C, Kirby S, Simner J, Hubbard EM (2013). Synaesthesia, cross-modality and language evolution. Oxford handbook of synaesthesia.

[CR60] Cytowic RE (2002). Synesthesia: a union of the senses.

[CR61] Cytowic RE, Eagleman DM (2009). Wednesday is Indigo blue: discovering the brain of synesthesia (with an afterword by Dmitri Nabokov).

[CR62] Cytowic RE (1993) The man who tasted shapes: a bizarre medical mystery offers revolutionary insights into emotions, reasoning, and consciousness. GP Putnam’s Sons, New York

[CR63] Dager SR, Wang L, Friedman SD, Shaw DW, Constantino JN, Artru AA, Dawson G, Csernansky JG (2007). Shape mapping of the hippocampus in young children with autism spectrum disorder. Am J Neuroradiol.

[CR64] Daniels EC, Rodriguez A, Zabelina DL (2020). Severity of misophonia symptoms is associated with worse cognitive control when exposed to misophonia trigger sounds. PLoS ONE.

[CR65] D'Argembeau A, Raffard S, Van der Linden M (2008). Remembering the past and imagining the future in schizophrenia. J Abnorm Psychol.

[CR66] David N, Gawronski A, Santos NS, Huff W, Lehnhardt FG, Newen A, Vogeley K (2008). Dissociation between key processes of social cognition in autism: Impaired mentalizing but intact sense of agency. J Autism Dev Disord.

[CR67] De Guzman M, Bird G, Banissy MJ, Catmur C (2016). Self–other control processes in social cognition: from imitation to empathy. Philos Trans Royal Soci B: Biol Sci.

[CR68] de Villiers PA, de Villiers JG (1974). On this, that, and the other: nonegocentrism in very young children. J Exp Child Psychol.

[CR69] Decety J, Chaminade T (2003). When the self represents the other: a new cognitive neuroscience view on psychological identification. Consc Cogn.

[CR70] Dehaene S (2014). Consciousness and the brain: deciphering how the brain codes our thoughts.

[CR71] Delevoye-Turrell Y, Vienne C, Coello Y (2011). Space boundaries in schizophrenia. Social Psychol.

[CR72] Dennett DC (1991). Consciousness explained.

[CR73] Di Francesco M, Marraffa M, Paternoster A (2016). The self and its defenses. From psychodynamics to cognitive science.

[CR74] Diehl JJ, Bennetto L, Young EC (2006). Story recall and narrative coherence of high-functioning children with autism spectrum disorders. J Abnorm Child Psychol.

[CR75] Ditman T, Kuperberg GR (2007). The time course of building discourse coherence in schizophrenia: an ERP investigation. Psychophysiology.

[CR76] Dodd S (2005). Understanding autism.

[CR77] Duff MC, Hengst JA, Tranel D, Cohen NJ (2008). Collaborative discourse facilitates efficient communication and new learning in amnesia. Brain Lang.

[CR78] Eddy CM (2018). Social cognition and self-other distinctions in neuropsychiatry: insights from schizophrenia and Tourette syndrome. Prog Neuropsychopharmacol Biol Psychiatry.

[CR79] Edelstein M, Brang D, Rouw R, Ramachandran VS (2013). Misophonia: physiological investigations and case descriptions. Front Hum Neurosci.

[CR80] Everett C (2013). Linguistic relativity: evidence across languages and cognitive domains.

[CR81] Ferretti F, Adornetti I, Chiera A, Nicchiarelli S, Valeri G, Magni R, Vicari S, Marini A (2018). Time and narrative: an investigation of storytelling abilities in children with autism spectrum disorder. Front Psychol.

[CR82] Ferri F, Frassinetti F, Mastrangelo F, Salone A, Ferro FM, Gallese V (2012). Bodily self and schizophrenia: the loss of implicit self-body knowledge. Conscious Cogn.

[CR83] Ferri F, Costantini M, Salone A, Di Iorio G, Martinotti G, Chiarelli A, Merla A, Di Giannantonio M, Gallese V (2014). Upcoming tactile events and body ownership in schizophrenia. Schizophr Res.

[CR84] Fineberg SK, Leavitt J, Deutsch-Link S, Dealy S, Landry CD, Pirruccio K, Shea S, Trent S, Cecchi G, Corlett PR (2016). Self-reference in psychosis and depression: a language marker of illness. Psychol Med.

[CR85] Fink A, Benedek M, Unterrainer HF, Papousek I, Weiss EM (2014). Creativity and psychopathology: are there similar mental processes involved in creativity and in psychosis-proneness?. Front Psychol.

[CR86] Finnegan EG, Asaro-Saddler K, Zajic MC (2021). Production and comprehension of pronouns in individuals with autism: a meta-analysis and systematic review. Autism.

[CR87] Fitzpatrick SE, Srivorakiat L, Wink LK, Pedapati EV, Erickson CA (2016). Aggression in autism spectrum disorder: presentation and treatment options. Neuropsychiatr Dis Treat.

[CR88] Fornara GA, Papagno C, Berlingeri M (2017). A neuroanatomical account of mental time travelling in schizophrenia: a meta-analysis of functional and structural neuroimaging data. Neurosci Biobehav Rev.

[CR89] Frith U (1989). Autism: explaining the enigma.

[CR90] Frith CD (1992). The cognitive neuropsychology of schizophrenia.

[CR91] Frith C (2002). Attention to action and awareness of other minds. Conscious Cogn.

[CR92] Frith U, De Vignemont F (2005). Egocentrism, allocentrism and asperger syndrome. Conscious Cogn.

[CR93] Frith CD, Done DJ (1989). Experiences of alien control in schizophrenia reflect a disorder in the central monitoring of action. Psychol Med.

[CR94] Frith U, Happé F (1999). Theory of mind and self-consciousness: what is it like to be autistic?. Mind Lang.

[CR95] Frith CD, Blakemore SJ, Wolpert DM (2000). Explaining the symptoms of schizophrenia: abnormalities in the awareness of action. Brain Res Rev.

[CR96] Gallagher S, Shear J (1999). Models of the Self.

[CR97] Gallagher S (2000). Philosophical conceptions of the self: implications for cognitive science. Trends Cogn Sci.

[CR98] Gallagher S, Zahavi D (2012). The phenomenological mind.

[CR99] Gallese V, Ferri F (2014). Psychopathology of the bodily self and the brain: the case of schizophrenia. Psychopathology.

[CR100] Garbarini F, Mastropasqua A, Sigaudo M, Rabuffetti M, Piedimonte A, Pia L, Rocca P (2016). Abnormal sense of agency in patients with schizophrenia: evidence from bimanual coupling paradigm. Front Behav Neurosci.

[CR101] Gärdenfors P (2000). Conceptual spaces: the geometry of thought.

[CR102] Gärdenfors P (2014). The geometry of meaning: Semantics based on conceptual spaces.

[CR103] Genette G (1972). Discours du Récit, Essai de Méthode.

[CR104] Giddens A (1991). Modernity and self-identity: self and society in the late modern age.

[CR105] Grossenbacher PG, Lovelace CT (2001). Mechanisms of synesthesia: cognitive and physiological constraints. Trends Cogn Sci.

[CR106] Habermas T, de Silveira C (2008). The development of global coherence in life narratives across adolescence: temporal, causal, and thematic aspects. Dev Psychol.

[CR107] Habermas T, Köber C, McLean KC, Syed MU (2014). Autobiographical reasoning is constitutive for narrative identity: the role of the life story for personal continuity. The Oxford handbook of identity development.

[CR108] Haggard P (2017). Sense of agency in the human brain. Nat Rev Neurosci.

[CR109] Hänggi J, Wotruba D, Jäncke L (2011). Globally altered structural brain network topology in grapheme-color synesthesia. J Neurosci.

[CR110] Happé F, Frith U (2006). The weak coherence account: detail-focused cognitive style in autism spectrum disorders. J Autism Dev Disord.

[CR111] Happé F, Frith U, Briskman J (2001). Exploring the cognitive phenotype of autism: weak “central coherence” in parents and siblings of children with autism: I Experimental tests. J Child Psychol Psychiat Allied Discipl.

[CR112] Hare B (2017). Survival of the friendliest: homo sapiens evolved via selection for prosociality. Annu Rev Psychol.

[CR113] Hare B, Woods V (2020). Survival of the friendliest: Understanding our origins and rediscovering our common humanity.

[CR114] Harrington L, Siegert R, McClure J (2005). Theory of mind in schizophrenia: a critical review. Cogn Neuropsychiatry.

[CR115] Heine B, Kuteva T (2007). The genesis of grammar.

[CR116] Henriksen MG, Nordgaard J (2014). Schizophrenia as a disorder of the self. J Psychopathol.

[CR117] Hill AP, Zuckerman KE, Hagen AD, Kriz DJ, Duvall SW, van Santen J, Nigg J, Fair D, Fombonne E (2014). Aggressive behavior problems in children with autism spectrum disorders: prevalence and correlates in a large clinical sample. Res Autism Spectr Disord.

[CR118] Hobson RP (1990). On the origins of self and the case of autism. Dev Psychopathol.

[CR119] Hobson RP, Lee A, Hobson JA (2010). Personal pronouns and communicative engagement in autism. J Autism Dev Disord.

[CR120] Hobson PR, Chidambi G, Lee A, Meyer J (2006) Foundations for self-awareness: an exploration through autism, Monogr Soc Res Child Dev 71:(2):vii–166. 10.1111/j.1540-5834.2006.00387.x 10.1111/j.1540-5834.2006.00387.x 17177915

[CR121] Hofmann W, Schmeichel BJ, Baddeley AD (2012). Executive functions and self-regulation. Trends Cogn Sci.

[CR122] Holle H, Banissy MJ, Ward J (2013). Functional and structural brain differences associated with mirror-touch synaesthesia. Neuroimage.

[CR123] Hopper PJ, Traugott EC (2003). Grammaticalization.

[CR124] Hubbard EM (2007). Neurophysiology of synesthesia. Curr Psychiatry Rep.

[CR125] Hubbard EM, Ramachandran VS (2005). Neurocognitive mechanisms of synesthesia. Neuron.

[CR126] Hur JW, Kwon JS, Lee TY, Park S (2014). The crisis of minimal self-awareness in schizophrenia: a meta-analytic review. Schizophr Res.

[CR127] Ioumpa K, Graham SA, Clausner T, Fisher SE, Van Lier R, Van Leeuwen TM (2019). Enhanced self-reported affect and prosocial behaviour without differential physiological responses in mirror-sensory synaesthesia. Philos Trans R Soc B.

[CR128] Jäncke L, Beeli G, Eulig C, Hänggi J (2009). The neuroanatomy of grapheme-color synesthesia. Eur J Neurosci.

[CR129] Jeannerod M (2009). The sense of agency and its disturbances in schizophrenia: a reappraisal. Exp Brain Res.

[CR130] Jeannerod M (2007) From my self to other selves: a revised framework for the self/other differentiation. Sensorimotor foundations of higher cognition, p 233–248

[CR131] Jolliffe T, Baron-Cohen S (2000). Linguistic processing in high-functioning adults with autism or Asperger's syndrome Is global coherence impaired?. Psychol Medicine.

[CR132] Jordan RR (1989). An experimental comparison of the understanding and use of speaker-addressee personal pronouns in autistic children. Int J Lang Commun Disord.

[CR133] Jordan R (2010). Autism spectrum disorders: an introductory handbook for practitioners.

[CR134] Kambanaros M, Christou N, Grohmann KK (2019). Interpretation of compound words by Greek-speaking children with autism spectrum disorder plus language impairment (ASD–LI). Clin Linguist Phon.

[CR135] Kanner L (1943). Autistic disturbances of affective contact. Nervous Child.

[CR136] Kerns JG (2007). Verbal communication impairments and cognitive control components in people with schizophrenia. J Abnorm Psychol.

[CR137] Kerns JG, Berenbaum H (2002). Cognitive impairments associated with formal thought disorder in people with schizophrenia. J Abnorm Psychol.

[CR138] King D, Dockrell J, Stuart M (2014). Constructing fictional stories: a study of story narratives by children with autistic spectrum disorder. Res Dev Disabil.

[CR139] Kinzler KD, Spelke ES (2007). Core systems in human cognition. Prog Brain Res.

[CR140] Kuang S (2016). Toward a unified social motor cognition theory of understanding mirror-touch synaesthesia. Front Hum Neurosci.

[CR141] Kuperberg G (2010). Language in schizophrenia part 1: an introduction. Lang Linguist Compass.

[CR142] Kutas M, Federmeier KD (2011). Thirty years and counting: finding meaning in the N400 component of the event-related brain potential (ERP). Annu Rev Psychol.

[CR143] Kutas M, Hillyard SA (1980). Reading senseless sentences: Brain potentials reflect semantic incongruity. Science.

[CR144] Lakoff G, Johnson M (1980). Metaphors we live by.

[CR145] Lee A, Hobson RP, Chiat S (1994). I, you, me, and autism: an experimental study. J Autism Dev Disord.

[CR146] Lewis M, Ramsay D (2004). Development of self-recognition, personal pronoun use, and pretend play during the 2nd year. Child Dev.

[CR147] Li Q, Cheung C, Wei R, Hui ES, Feldon J, Meyer U, Chung S, Chua SE, Sham PC, Wu EX, McAlonan GM (2009). Prenatal immune challenge is an environmental risk factor for brain and behavior change relevant to schizophrenia: evidence from MRI in a mouse model. PLoS ONE.

[CR148] Liepelt R, Schneider JC, Aichert DS, Wöstmann N, Dehning S, Möller HJ, Riedel M, Dolk T, Ettinger U (2012). Action blind: disturbed self-other integration in schizophrenia. Neuropsychologia.

[CR149] Lombardo MV, Baron-Cohen S (2010). Unraveling the paradox of the autistic self. Wiley Interdisc Rev: Cognit Sci.

[CR150] Lombardo MV, Barnes JL, Wheelwright SJ, Baron-Cohen S (2007). Self-referential cognition and empathy in autism. PLoS ONE.

[CR151] Lombardo MV, Chakrabarti B, Bullmore ET, Sadek SA, Pasco G, Wheelwright SJ, Baron-Cohen S (2010). Atypical neural self-representation in autism. Brain.

[CR152] Losh M, Capps L (2003). Narrative ability in high-functioning children with autism or Asperger's syndrome. J Autism Dev Disord.

[CR153] Lyons AD, Henry JD, Rendell PG, Robinson G, Suddendorf T (2016). Episodic foresight and schizophrenia. Br J Clin Psychol.

[CR154] Lyons V, Fitzgerald M (2013) Atypical sense of self in autism spectrum disorders: a neuro-cognitive perspective. In: Recent advances in autism spectrum disorders-Volume I. IntechOpen

[CR155] Lysaker PH, Lysaker JT (2010). Schizophrenia and alterations in self-experience: a comparison of 6 perspectives. Schizophr Bull.

[CR156] Lysaker PH, Olesek KL, Warman DM, Martin JM, Salzman AK, Nicolò G, Salvatore G, Dimaggio G (2011). Metacognition in schizophrenia: correlates and stability of deficits in theory of mind and self-reflectivity. Psychiatry Res.

[CR157] MacKay DG, Burke DM, Stewart R (1998). HM's language production deficits: implications for relations between memory, semantic binding, and the hippocampal system. J Memory Lang.

[CR158] MacKay DG, James LE, Taylor JK, Marian DE (2007). Amnesic H.M. exhibits parallel deficits and sparing in language and memory: systems versus binding theory accounts. Lang Cognit Process.

[CR159] Mackinley M, Chan J, Ke H, Dempster K, Palaniyappan L (2021). Linguistic determinants of formal thought disorder in first episode psychosis. Early Interv Psychiatry.

[CR160] Maguire EA, Burgess N, Donnett JG, Frackowiak RS, Frith CD, O’Keefe J (1998). Knowing where and getting there: a human navigation network. Science.

[CR161] Maguire EA, Intraub H, Mullally SL (2016). Scenes, spaces, and memory traces: what does the hippocampus do?. Neuroscientist.

[CR162] Maister L, Banissy MJ, Tsakiris M (2013). Mirror-touch synaesthesia changes representations of self-identity. Neuropsychologia.

[CR163] Malek HB, D’Argembeau A, Allé MC, Meyer N, Danion JM, Berna F (2019). Temporal processing of past and future autobiographical events in patients with schizophrenia. Sci Rep.

[CR164] Marini A, Spoletini I, Rubino IA, Ciuffa M, Bria P, Martinotti G, Banfi G, Boccascino R, Strom P, Siracusano A, Caltagirone C (2008). The language of schizophrenia: an analysis of micro and macrolinguistic abilities and their neuropsychological correlates. Schizophr Res.

[CR165] Marini A, Ferretti F, Chiera A, Magni R, Adornetti I, Nicchiarelli S, Vicari S, Valeri G (2019). Episodic future thinking and narrative discourse generation in children with Autism Spectrum Disorders. J Neuroling.

[CR166] Marini A, Ozbič M, Magni R, Valeri G (2020). Toward a definition of the linguistic profile of children with autism spectrum disorder. Front Psychol.

[CR167] Maurer D, de Boysson-Bardies B, de Schonen S, Jusczyk PW, McNeilage P, Morton J (1993). Neonatal synesthesia: implications for the processing of speech and faces. NATO ASI series D: behavioural and social sciences.

[CR168] Maurer D, Mondloch CJ (2006). The infant as synesthete. Attent Perform.

[CR169] McAdams DP (2001). The psychology of life stories. Rev Gen Psychol.

[CR170] McAdams DP, McLean KC (2013). Narrative identity. Curr Dir Psychol Sci.

[CR171] McCabe A, Hillier A, Shapiro C (2013). Brief report: Structure of personal narratives of adults with autism spectrum disorder. J Autism Dev Disord.

[CR172] McKenna PJ, Oh TM (2005). Schizophrenic speech: making sense of bathroots and ponds that fall in doorways.

[CR173] Mead GH (1934). Mind, self, and society (CW Morris).

[CR174] Mehling WE, Gopisetty V, Daubenmier J, Price CJ, Hecht FM, Stewart A (2009). Body awareness: construct and self-report measures. PLoS ONE.

[CR175] Meltzoff AN, Cicchetti D, Beeghly M (1990). Foundations for developing a concept of self: The role of imitation in relating self to other and the value of social mirroring, social modeling, and self practice in infancy. The self in transition: infancy to childhood.

[CR176] Michael J, Park S (2016). Anomalous bodily experiences and perceived social isolation in schizophrenia: an extension of the social deafferentation hypothesis. Schizophr Res.

[CR177] Milivojevic B, Varadinov M, Grabovetsky AV, Collin SH, Doeller CF (2016). Coding of event nodes and narrative context in the hippocampus. J Neurosci.

[CR178] Milne E, Szczerbinski M (2009). Global and local perceptual style, field-independence, and central coherence: an attempt at concept validation. Adv Cogn Psychol.

[CR179] Moyer M, Harrigan K, Hacquard V, Lidz J (2015) 2-year-olds’ comprehension of personal pronouns. In: Online supplemental proceedings of the 39th Boston university conference on language development

[CR180] Nasrallah HA (2012). Impaired mental proprioception in schizophrenia. Editorial Curr Psych.

[CR181] Neisser U (1988). Five kinds of self-knowledge. Philos Psychol.

[CR182] Nelson B, Fornito A, Harrison BJ, Yücel M, Sass LA, Yung AR, Thompson A, Wood SJ, Pantelis C, McGorry PD (2009). A disturbed sense of self in the psychosis prodrome: linking phenomenology and neurobiology. Neurosci Biobehav Rev.

[CR183] Nestor P, Akdag S, O’Donnell B, Niznikiewicz M, Law S, Shenton M, McClarley R (1998). Word recall in schizophrenia: a connectionist model. Am J Psychiatry.

[CR184] Nieuwland MS, Van Berkum JJ (2006). When peanuts fall in love: N400 evidence for the power of discourse. J Cogn Neurosci.

[CR185] Noel J-P, Cascio CJ, Wallace MT, Park S (2017). The spatial self in schizophrenia and autism spectrum disorder. Schizophr Res.

[CR186] Novogrodsky R (2013). Subject pronoun use by children with autism spectrum disorders (ASD). Clin Linguist Phon.

[CR187] Novogrodsky R, Edelson LR (2016). Ambiguous pronoun use in narratives of children with Autism spectrum disorders. Child Lang Teach Therapy.

[CR188] Nuske HJ, Bavin EL (2011). Narrative comprehension in 4–7-year-old children with autism: testing the weak central coherence account. Int J Lang Commun Disord.

[CR189] O'Dowd A, Cooney SM, McGovern DP, Newell FN (2019). Do synaesthesia and mental imagery tap into similar cross-modal processes?. Philos Trans R Soc B.

[CR190] Palaniyappan L (2021). More than a biomarker: could language be a biosocial marker of psychosis?. NPJ Schizophr.

[CR191] Paton B, Hohwy J, Enticott PG (2012). The rubber hand illusion reveals proprioceptive and sensorimotor differences in autism spectrum disorders. J Autism Dev Disord.

[CR192] Pawełczyk A, Kotlicka-Antczak M, Łojek E, Ruszpel A, Pawełczyk T (2018). Schizophrenia patients have higher-order language and extralinguistic impairments. Schizophr Res.

[CR193] Peled A, Ritsner M, Hirschmann S, Geva AB, Modai I (2000). Touch feel illusion in schizophrenic patients. Biol Psychiat.

[CR194] Peled A, Pressman A, Geva AB, Modai I (2003). Somatosensory evoked potentials during a rubber-hand illusion in schizophrenia. Schizophr Res.

[CR195] Pierce JP (2018). Transthesia: comparing the prevalence of synesthesia in transgender and cisgender individuals. Transg Health.

[CR196] Progovac L, Benítez-Burraco A (2019). From physical aggression to verbal behavior: language evolution and self-domestication feedback loop. Front Psychol.

[CR197] Quoidbach J, Hansenne M, Mottet C (2008). Personality and mental time travel: a differential approach to autonoetic consciousness. Conscious Cogn.

[CR198] Race E, Keane MM, Verfaellie M (2015). Sharing mental simulations and stories: hippocampal contributions to discourse integration. Cortex.

[CR199] Radvansky GA, Zacks JM (2014). Event cognition.

[CR200] Raffard S, Esposito F, Boulenger JP, Van der Linden M (2013). Impaired ability to imagine future pleasant events is associated with apathy in schizophrenia. Psychiatry Res.

[CR201] Ramachandran VS, Hubbard EM (2001). Synaesthesia–a window into perception, thought and language. J Conscious Stud.

[CR202] Rapp AM, Felsenheimer AK, Langohr K, Klupp M (2018). The comprehension of familiar and novel metaphoric meanings in schizophrenia: a pilot study. Front Psychol.

[CR203] Renes RA, Vermeulen L, Kahn RS, Aarts H, van Haren NE (2013). Abnormalities in the establishment of feeling of self-agency in schizophrenia. Schizophr Res.

[CR204] Rentzeperis I, van Leeuwen C (2021). Adaptive rewiring in weighted networks shows specificity, robustness, and flexibility. Front Syst Neurosci.

[CR205] Riches NG, Loucas T, Baird G, Charman T, Simonoff E (2012). Interpretation of compound nouns by adolescents with specific language impairment and autism spectrum disorders: An investigation of phenotypic overlap. Int J Speech Lang Pathol.

[CR206] Rochester S, Martin JR (1979). Crazy talk: a study of the discourse of schizophrenic speakers.

[CR207] Rothen N, Meier B (2013). Why vicarious experience is not an instance of synesthesia. Front Hum Neurosci.

[CR208] Rouw R, Scholte HS (2007). Increased structural connectivity in grapheme-color synesthesia. Nat Neurosci.

[CR209] Rouw R, Scholte HS, Colizoli O (2011). Brain areas involved in synaesthesia: a review. J Neuropsychol.

[CR210] Roy P, Chiat S, Marshall CR (2013). Teasing apart disadvantage from disorder: the case of poor language. Current issues in developmental disorders.

[CR211] Russell J (1996). Agency: its role in mental development.

[CR212] Russell J, Hill EL (2001). Action-monitoring and intention reporting in children with autism. J Child Psychol Psychiatry.

[CR213] Russell J, Jarrold C (1999). Memory for actions in children with autism: self versus other. Cogn Neuropsychiatry.

[CR214] Sah WH, Torng PC (2015). Narrative coherence of Mandarin-speaking children with high-functioning autism spectrum disorder: an investigation into causal relations. First Lang.

[CR215] Santiesteban I, Bird G, Tew O, Cioffi MC, Banissy MJ (2015). Mirror-touch synaesthesia: difficulties inhibiting the other. Cortex.

[CR216] Schechtman M (1996). The constitution of selves.

[CR217] Schröder A, Vulink N, Denys D (2013). Misophonia: diagnostic criteria for a new psychiatric disorder. PLoS ONE.

[CR218] Schröder A, van Wingen G, Eijsker N (2019). Misophonia is associated with altered brain activity in the auditory cortex and salience network. Sci Rep.

[CR219] Shield A, Meier RP, Tager-Flusberg H (2015). The use of sign language pronouns by native-signing children with autism. J Autism Dev Disord.

[CR220] Silbersweig DA, Stern E, Frith C, Cahill C, Holmes A, Grootoonk S, Seaward J, McKenna P, Chua SE, Frackowiak RSJ (1995). A functional neuroanatomy of hallucinations in schizophrenia. Nature.

[CR221] Sinha C, Gärdenfors P (2014). Time, space, and events in language and cognition: a comparative view. Ann NY Acad Sci.

[CR222] Smith ADM, Höfler S, Díaz-Vera JE (2014). The pivotal role of metaphor in the evolution of human language. Metaphor and metonomy across time and cultures: perspectives on the sociohistorical linguistics of figurative language.

[CR223] Sowden S, Shah P (2014). Self-other control: a candidate mechanism for social cognitive function. Front Hum Neurosci.

[CR224] Speer NK, Zacks JM, Reynolds JR (2007). Human brain activity time-locked to narrative event boundaries. Psychol Sci.

[CR225] Spelke ES (2000). Core knowledge. Am Psychol.

[CR226] Spelke E, Gentner D, Goldin-Meadow S (2003). What makes us smart?. Language in Mind.

[CR227] Stefanatos GA, Baron IS (2011). The ontogenesis of language impairment in autism: a neuropsychological perspective. Neuropsychol Rev.

[CR228] Stephane M, Kuskowski M, McClannahan K, Surerus C, Nelson K (2010). Evaluation of speech misattribution bias in schizophrenia. Psychol Med.

[CR229] Stephane M, Kuskowski M, Gundel J (2014). Abnormal dynamics of language in schizophrenia. Psychiatry Res.

[CR230] Stern DN (1985). The interpersonal world of the infant.

[CR231] Sterponi L, de Kirby K, Shankey J, O’Reilly M, Lester JN (2015). Subjectivity in Autistic language: insights on pronoun atypicality from three case studies. The palgrave handbook of child mental health.

[CR232] Sterzer P, Adams RA, Fletcher P, Frith C, Lawrie SM, Muckli L, Petrovic P, Uhlhaas P, Voss M, Corlett PR (2018). The predictive coding account of psychosis. Biol Psychiat.

[CR233] Stirling L, Douglas S, Leekam S, Carey L, Arciuli J, Brock J (2014). The use of narrative in studying communication in autism spectrum disorders. Communication in autism.

[CR234] Surguladze S, Rossell S, Rabe-Hesketh S, David AS (2002). Cross-modal semantic priming in schizophrenia. J Int Neuropsychol Soc.

[CR235] Tager-Flusberg H, Paul P, Lord C, Volkmar FR, Klin A, Paul R, Cohen DJ (2005). Language and communication in autism. Handbook of autism and pervasive developmental disorders.

[CR236] Tang SX, Kriz R, Cho S, Park SJ, Harowitz J, Gur RE, Bhati MT, Wolf DH, Sedoc J, Liberman MY (2021). Natural language processing methods are sensitive to sub-clinical linguistic differences in schizophrenia spectrum disorders. NPJ Schizophr.

[CR237] Thakkar KN, Nichols HS, McIntosh LG, Park S (2011). Disturbances in body ownership in schizophrenia: evidence from the rubber hand illusion and case study of a spontaneous out-of-body experience. PLoS ONE.

[CR238] Thomas J, Kirby S (2018). Self domestication and the evolution of language. Biol Philos.

[CR239] Thomas MS, Davis R, Karmiloff-Smith A, Knowland VC, Charman T (2016). The over-pruning hypothesis of autism. Dev Sci.

[CR240] Tordjman S, Celume MP, Denis L, Motillon T, Keromnes G (2019). Reframing schizophrenia and autism as bodily self-consciousness disorders leading to a deficit of theory of mind and empathy with social communication impairments. Neurosci Biobehav Rev.

[CR241] Trabasso T, Sperry LL (1985). Causal relatedness and importance of story events. J Mem Lang.

[CR242] Tsakiris M (2008). Looking for myself: current multisensory input alters self-face recognition. PLoS ONE.

[CR243] Tsakiris M, Hesse MD, Boy C, Haggard P, Fink GR (2007). Neural signatures of body ownership: a sensory network for bodily self-consciousness. Cereb Cortex.

[CR244] Uddin LQ, Iacoboni M, Lange C, Keenan JP (2007). The self and social cognition: the role of cortical midline structures and mirror neurons. Trends Cogn Sci.

[CR245] Van den Berg D, Gong P, Breakspear M, van Leeuwen C (2012). Fragmentation: loss of global coherence or breakdown of modularity in functional brain architecture?. Front Syst Neurosci.

[CR246] Van Leeuwen TM, Van Petersen E, Burghoorn F, Dingemanse M, Van Lier R (2019). Autistic traits in synaesthesia: atypical sensory sensitivity and enhanced perception of details. Philos Trans R Soc B.

[CR247] van Os J, Kapur S (2009). Schizophrenia. Lancet.

[CR248] van Leeuwen TM, Neufeld J, Hughes J, Ward J (2020). Synaesthesia and autism: different developmental outcomes from overlapping mechanisms?. Cogn Neuropsychol.

[CR249] Vinogradova OS (2001). Hippocampus as comparator: role of the two input and two output systems of the hippocampus in selection and registration of information. Hippocampus.

[CR250] Ward J (2013). Synesthesia. Annu Rev Psychol.

[CR251] Ward J, Hoadley C, Hughes JE, Smith P, Allison C, Baron-Cohen S, Simner J (2017). Atypical sensory sensitivity as a shared feature between synaesthesia and autism. Sci Rep.

[CR252] Watanabe T, Rees G (2016). Anatomical imbalance between cortical networks in autism. Sci Rep.

[CR253] Webster L (2019). “I am I”: self-constructed transgender identities in internet-mediated forum communication. Int J Sociol Lang.

[CR254] Williams D, Happé F (2009). Pre-conceptual aspects of self-awareness in autism spectrum disorder: the case of action-monitoring. J Autism Dev Disord.

[CR255] Willits JA, Rubin T, Jones MN, Minor KS, Lysaker PH (2018). Evidence of disturbances of deep levels of semantic cohesion within personal narratives in schizophrenia. Schizophr Res.

[CR256] Witkin HA, Dyk RB, Faterson HF, Goodenough DR, Karp S (1962). Psychological differentiation: studies of development.

[CR257] Woo TU (2014). Neurobiology of schizophrenia onset. Curr Top Behav Neurosci.

[CR258] Yang ZY, Xie DJ, Zou YM, Wang Y, Li Y, Shi HS, Zhang RT, Li WX, Cheung EF, Kring AM, Chan RC (2018). Prospection deficits in schizophrenia: evidence from clinical and subclinical samples. J Abnorm Psychol.

[CR259] Zacks JM, Speer NK, Reynolds JR (2009). Segmentation in reading and film comprehension. J Exp Psychol Gen.

[CR260] Zahavi D, Fuchs T, Sattel H, Heningsen P (2010). Minimal self and narrative self. a distinction in need of refinement. The embodied self dimensions, coherence and disorders.

[CR261] Zahavi D (2014). Self and Other: Exploring Subjectivity, Empathy, and Shame.

[CR262] Zalla T, Verlut I, Franck N, Puzenat D, Sirigu A (2004). Perception of dynamic action in patients with schizophrenia. Psychiat Res.

[CR263] Zalla T, Bouchilloux N, Labruyere N, Georgieff N, Bougerol T, Franck N (2006). Impairment in event sequencing in disorganised and non-disorganised patients with schizophrenia. Brain Res Bull.

[CR264] Zhou Y, Shu N, Liu Y, Song M, Hao Y, Liu H, Yu C, Liu Z, Jiang T (2008). Altered resting-state functional connectivity and anatomical connectivity of hippocampus in schizophrenia. Schizophr Res.

